# Prophylactic dressings for preventing nasal pressure ulcer in premature newborns: an effectiveness review

**DOI:** 10.1590/1980-220X-REEUSP-2025-0099en

**Published:** 2025-11-28

**Authors:** Cibelle Mello Viero, Paulo Jorge Pereira Alves, Oclaris Lopes Munhoz, Thaynan Silveira Cabral, Cristina Maria Galvão, Valdecir Zavarese da Costa, Thaís Dresch Eberhardt, Matheus Silvelo Franco, Suzinara Beatriz Soares de Lima

**Affiliations:** 1 Universidade Federal de Santa Maria, Centro de Ciências da Saúde, Programa de Pós-Graduação de Enfermagem, Santa Maria, RS, Brazil.; 2 Universidade Católica Portuguesa, Faculdade de Ciências da Saúde e Enfermagem, Porto, Portugal.; 3 Universidade Federal de Santa Maria, Departamento de Ciências da Saúde, Palmeira das Missões, RS, Brazil.; 4 Universidade de São Paulo, Escola de Enfermagem, Ribeirão Preto, SP, Brazil.; 5 Universidade Federal de Santa Maria, Centro de Ciências da Saúde, Departamento de Enfermagem, Santa Maria, RS, Brazil.; 6 Universidade de Passo Fundo, Passo Fundo, RS, Brazil.

**Keywords:** Pressure Ulcer, Infant, Newborn, Intensive Care Units, Neonatal, Systematic Review, Evidence-Based Practice

## Abstract

**Objective:**

To evaluate the effectiveness of prophylactic dressings for preventing nasal pressure ulcer in newborns using respiratory medical devices.

**Method:**

Systematic review of effectiveness, according to the JBI methodology. Studies involving newborns using respiratory medical devices who received prophylactic dressing intervention were included. Descriptive synthesis and network meta-analysis were performed. Registration PROSPERO CRD42024516296.

**Results:**

Twelve studies were included, with a total of 1,001 newborns. The use of 1.8 mm silicone reduces the risk of nasal pressure ulcer, compared to paraffin oil (RR 0.13; 95% CI = 0.02, 0.89), nasal plug (RR 0.13; 95% CI = 0.03, 0.53), and not intervening (RR 0.29; 95% CI = 0.09, 0.85), as well as the use of hydrocolloid reduces the risk of injury when compared to the plug (RR 0.28; 95% CI = 0.14, 0.56) and not intervening (RR 0.60; 95% CI = 0.38, 0.93).

**Conclusion:**

Prophylactic dressings, especially 1.8 mm silicone gel and hydrocolloid, are effective in preventing nasal pressure ulcers in premature newborns using respiratory devices.

## INTRODUCTION

It should be highlighted that the structure of premature infants’ skin is qualitatively and quantitatively distinct from the skin of full-term newborns and infants. Furthermore, the mechanisms of tissue damage in this population present unique characteristics^(1)^. In this context, newborns (NB) admitted to a neonatal intensive care unit (NICU) are at risk of developing skin damage, especially pressure ulcers (PU), which is a problem that deserves the health professionals attention. PUs related to medical devices are among the most frequent, reaching 73.5%, mainly associated with continuous positive airway pressure (CPAP)^(2)^.

Prolonged use of ventilation masks, such as CPAP, exposes facial soft tissues to an increased risk of rupture due to continuous deformation of the skin and underlying tissues at the contact interface with these devices^(3,4)^.

Although the use of prophylactic dressings is recommended in international clinical guidelines and is considered standard of care in some clinical contexts, there is still no standardization or consensus among researchers to evaluate the preventive function of these materials^(5)^. However, it is known that the main requirement of this type of coverage is the reduction of localized and supported tissue loads on the soft tissues, between the device and the skin^(6)^.

The application of certain skin protective barriers, such as hydrocolloids and foams, at the interface between the skin and the respiratory support system, such as the ventilatory therapy mask, can minimize the risk of PU by redistributing localized contact forces and dissipating surface and internal tensions in the tissues. Therefore, healthcare professionals must adopt objective, standardized, and evidence-based approaches in clinical practice decision-making to select the most effective products for preventing this condition^(4)^.

In view of the above, a preliminary search was carried out in the following information sources: International Prospective Register of Systematic Reviews (PROSPERO), MEDLINE, Cochrane Database of Systematic Reviews and JBI Collaboration, and no current or ongoing systematic reviews were identified on the effectiveness of prophylactic dressings for the prevention of nasal PU in newborns. A protocol was found with the objective of evaluating the evidence regarding preventive care for PU from nasal CPAP in premature newborns^(7)^ and another review that investigated the benefits of hydrocolloid dressing in preventing nasal tissue damage in babies using CPAP, with limitations on the year of publication and language^(8)^. Thus, it is pertinent to seek, evaluate, and synthesize evidence on the topic.

By gathering and critically analyzing the available evidence, this study aims to contribute to the qualification of neonatal nursing care, providing scientific support for the development of more effective and evidence-based care protocols. This way, we seek to enhance the safety and quality of care provided to newborns, reducing the occurrence of nasal PU and its impacts on the health of this vulnerable population. Thus, the objective of the study was to evaluate the effectiveness of prophylactic dressings for the prevention of nasal PU in newborns using a respiratory medical device, compared to a control group, placebo or usual care.

## METHOD

### Study Type, Protocol and Registration

Systematic review of effectiveness with network meta-analysis developed according to the JBI methodology^(9)^ and reported in accordance with the Preferred Reporting Items for Systematic Reviews and Meta-Analyses (PRISMA)^(10)^. Review protocol registered on the PROSPERO platform (CRD42024516296).

The review question was structured based on the acronym PICO, in which P (population) = newborns using a respiratory medical device; I (intervention) = prophylactic dressings; C (comparator) = control group, placebo or usual care; and O (outcome) = occurrence of nasal pressure ulcer. Therefore, the question is: what is the effectiveness of prophylactic dressings for preventing nasal PU in newborns using respiratory medical devices, compared to the control group, placebo or usual care?

### Eligibility Criteria

Experimental and observational studies with the participation of newborns^(11)^ hospitalized and using respiratory medical devices (equipment or its parts that were in direct contact with the skin for the administration of ventilatory therapy)^(12)^ were included. The patients received intervention with prophylactic dressings with gels or foams, such as silicone, hydrocolloid, polyurethane and polyvinyl, applied to the skin before the occurrence of tissue damage^(13)^. Furthermore, investigations comparing the intervention with non-standardized care or no intervention to placebo, oils, or usual care were included.

Studies involving NB with nasal PU or whose focus was investigating the use of dressings to treat this type of injury were excluded. No language delimitation or time frame was defined and duplicate productions were analyzed only once.

### Information Sources and Sampling

Searches for primary studies were conducted in April 2024, using the following information sources: Virtual Health Library (VHL), Medical Literature Analysis and Retrieval System Online (MEDLINE), via Pubmed, Scientific Electronic Library Online (SciELo) and Cochrane Library, EMBASE and Scopus (Elsevier), Web of Science (Clarivate), Academic Search Premier, Cumulative Index to Nursing and Allied Health Literature (CINAHL) with Full Text (EBSCO), SCIENCE and La Reference. A complementary search was also conducted on the website Google Scholar using the titles and authors of the studies included in the review.

Advanced search strategies were implemented, considering the specificities of each information source. Thus, the vocabularies, Health Sciences Descriptors (DeCS), synonyms, Medical Subject Headings (MESH), entry terms and CINAHL headings were used, which were combined with Boolean operators AND and OR. A librarian, with expertise in developing search strategies for conducting knowledge synthesis methods, reviewed the strategies developed for the different sources of information.

Subsequently, the search strategy delimited for the MEDLINE database was presented, namely: (((“Pressure Ulcer”[mh] OR Bed Sore* OR Bedsore* OR Decubitus Ulcer* OR Pressure Sore* OR Pressure Ulcer* OR decubital ulcer* OR decubitus ulceration OR pressure injur* OR pressure sore OR decubitus OR Nasal injuries OR “Nasal Septum”[mh] OR Nasal Septum* Nasal OR Nose[mh] OR Nose* OR Friction[mh] OR Friction OR Pressure[mh] OR Pressure*) AND (“Infant, Newborn”[mh] OR Newborn Infant* OR Newborn* OR Newborn* OR Infant[mh] OR Infant* OR “Child, Preschool”[mh] OR Preschool* OR Child[mh] OR Children OR “full term infant” OR neonatus OR “newly born baby” OR “newly born child” OR “newly born infant” OR Pediatric* OR “Infant, Premature”[mh] OR Preterm* OR Premature* OR “Neonatal Prematurity” OR prematurita* OR Neonatology[mh] OR Neonatolog*)) AND (“Continuous Positive Airway Pressure”[mh] OR Airway Pressure Release Ventilation OR APRV Ventilation Mode OR Bilevel Continuous Positive Airway Pressure OR Bilevel Positive Airway Pressure OR BiPAP OR Bilevel Positive Airway Pressure OR Biphasic Positive Airway Pressure OR Biphasic Continuous Positive Airway Pressure OR Biphasic Positive Airway Pressure OR CPAP Ventilation OR CPAP OR Nasal Continuous Positive Airway Pressure OR nCPAP Ventilation OR “Noninvasive Ventilation”[mh] OR Non Invasive Ventilation* OR Non-Invasive Ventilation* OR “Noninvasive Ventilations” OR Device* OR Medical Device* OR “Medical device-related pressure injuries” OR “Positive-Pressure Respiration”[mh] OR Positive End Expiratory Pressure OR Positive End-Expiratory Pressure* OR Positive Pressure Respiration OR Positive Pressure Ventilation OR Positive-Pressure Respirations OR Positive-Pressure Ventilation* OR “Respiration, Artificial”[mh] OR Artificial Respiration* OR Mechanical Ventilation* OR NCPAP OR “high flow nasal cannula”)) AND (Bandages[mh] OR Bandage OR Dressing* OR “Bandages, Hydrocolloid”[mh] OR Duoderm OR Hydrocolloid Bandage* OR Hydrocolloid Dressing* OR Hydrogel Bandage* OR “wound care device” OR “wound care product” OR “wound therapy device” OR “bandages and dressings” OR “Comfeel Plus” OR hydrocolloid OR duoderm OR Exuderm OR granuflex OR Tegasorb OR Varihesive OR Prophylactic dressing* OR Preventive dressing* OR Protective dressing* OR Preventive wound management OR Silicone dressings OR Silicone bandages OR Silicone wound dressings OR Silicone wound covers Polyvinyl dressings OR Polyvinyl bandages OR Polyvinyl wound dressings OR Polyvinyl wound covers OR “prevention & control” OR “preventive therapy”). Based on this strategy, adaptations for other sources of information were made. Due to the limited number of pages in the manuscript, it was not possible to fully include the search strategies for the different sources of information; however, they are available in the systematic review protocol record on the PROSPERO platform.

### Screening and Selection of Studies

All identified citations were grouped and loaded into the software *EndNote* (*Clarivate,* PA, USA), duplicates removed and imported with citation details into the software Rayyan: Systematic Review Management^(14)^, to carry out screening and selection.

The selection of primary studies was conducted by two reviewers, blindly and independently. The process began with the reading of titles and abstracts, followed by a complete analysis of the selected texts, according to the eligibility criteria. The reviewers then compared their results to find discrepancies, resolving them by consensus. The participation of a third reviewer was planned in cases of disagreement; however, it was not necessary.

### Methodological Quality Assessment

Instruments standardized by the JBI Collaboration for randomized clinical trials, cohort and case-control studies were used. These tools are checklists that have, respectively, thirteen, eleven and ten items (questions), with answer options of yes, no, uncertain/unclear or not applicable^(9)^.

Eligible studies were critically evaluated by two independent reviewers and disagreements were resolved with the assistance of a third reviewer. All studies, regardless of their methodological quality, underwent data extraction and synthesis.

### Data Extraction

The following data were extracted: study (authors, year, language, country, study design, setting, type of instrument/data collection) and sample (number of newborns, gestational age and birth weight, sex, type of respiratory medical device) characteristics, type of intervention in the groups, duration of intervention use, main results and conclusions related to nasal PU (incidence/prevalence of lesions in different groups – statistical differences, classification of lesions into stages). The collected data were organized into tables in Google Docs and later imported into *Microsoft Word*® and the outcomes included in the *Microsoft Excel*® software by one reviewer, with independent cross-checking by the other.

It should be noted that, in six studies included^(15, 16, 17, 18, 19, 20)^ there was not all the information required for data extraction and, at that time, the authors were contacted via email and social media *ResearchGate,* with a waiting time of 45 days; only one^(15)^ made the requested results available.

### Evidence Synthesis

All included studies were considered for evidence synthesis. For observational studies, a descriptive summary was prepared, using absolute (n) and relative (%) frequencies.

For experimental studies, a network meta-analysis was performed (*Network Meta-Analysis* – *NMA*), of random effects model, with direct and indirect comparisons of experimental studies^(21)^. The principle of transitivity was also considered, which requires that the competing interventions of a systematic review be randomizable together^(22)^ and, as a measure of effect, the Relative Risk (RR). The meta-analysis was conducted using CINeMA (Confidence in network meta-analysis), linked to the software R^®^, being especially used to calculate the contribution of studies in the relative effects^(21,22)^. The league table was taken from the software CINeMA.

Although the review was planned to include newborns in general, all studies eligible for the NMA exclusively evaluated preterm newborns. Thus, the statistical synthesis was conducted as a subgroup analysis, restricted to this population.

### Assessment of the Certainty of Evidence

The software Webapp from CINeMA was used for assessing the certainty of evidence, which covers six domains: risk of bias in the studies analyzed, reporting/publication bias, indirect evidence, imprecision, heterogeneity, and incoherence. For each domain, three levels of concern are assigned: “no concerns”, “some concerns” and “major concerns”, except for the “reporting bias” domain, which is classified as “low risk”, “some concerns” and “high risk”. The certainty of evidence can be categorized as “very low”, “low”, “moderate” or “high”, and in this review the outcome was attributed to the occurrence of nasal PU^(21,22)^.

## RESULTS

### Inclusion of Studies

**Figure 1:**
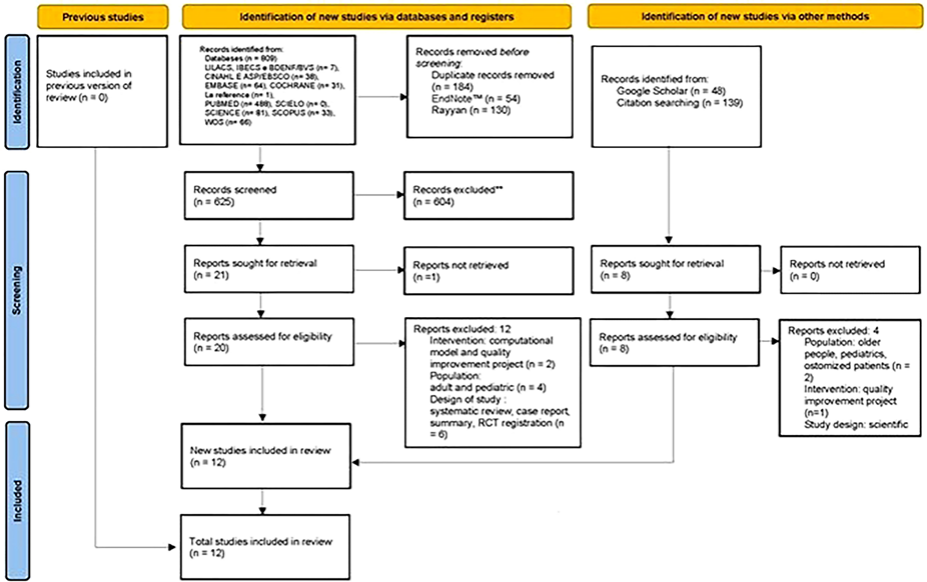
Study selection flowchart – Preferred Reporting Items for Systematic Review and Meta-Analyses (PRISMA)^(10)^.

A total of 809 records were identified in the information sources, of which 184 were duplicates, leaving 625 publications for reading titles and abstracts. Of these, 604 were excluded for not meeting the selection criteria. At the eligibility stage, 21 publications were read in full and 12 remained for evidence synthesis. A detailed flowchart of study selection was presented in Figure 1.

### Characterization of Included Studies

Regarding the study design, seven (53%) were randomized clinical trials (RCTs)^(15,16,20,23, 24, 25, 26)^, four (33%) in a cohort study^(17, 18, 19,27)^, and one (8.3%), a case-control study^(28)^.

Of the 12 studies included, one (8.3%) was from Turkey^(15)^, two (16.7%) from China^(20,23)^, two (16.7%) from Australia^(16,24)^, one (8.3%) from the United States of America^(17)^, two (16.7%) from Brazil^(25,28)^, one (8.3%) from Spain^(18)^, one (8.3%) from Lebanon^(27)^, one (8.3%) from Iran^(26)^, and one (8.3%) from Vietnam^(19)^. The total sample was 1,001 newborns (sum of all samples from the included studies), the publication period ranged from 2010 to 2021, 11 (91.7%) were published in English, and one (8.3%) in Spanish^(18)^.

Regarding the type of respiratory medical device and its interfaces: four (33.3%) studies used only nasal CPAP^(20)^ with nasal prongs^(15,23,26)^; one (8.3%) used nasal cannula for non-invasive ventilation (NIV)^(19)^; two (16.7%) NIV through short binasal prongs^(25,28)^; one (8.3%), CPAP through binasal prongs and heated humidified high-flow nasal cannulae (HHHFNC)^(16)^; one (8.3%), nasal cannula via CPAP and/or intermittent positive airway pressure ventilation in the nasal airways^(27)^; one (8.3%), binasal prongs via CPAP or nasal intermittent positive airway pressure ventilation^(24)^; one (8.3%), only prongs for high-flow humidified and heated nasal cannula^(17)^; one (8.3%) used NIV via high-flow oxygen therapy (HFO) and/or CPAP/Bilevel Positive Airway Pressure (BiPAP), via RAM cannula^®^, nasal rods, nasal mask, and nasal goggles^(18)^.

Regarding the interventional material tested, in six studies (50%), the authors applied the hydrocolloid dressing^(16,17,20,23,24,26)^; in one (8.3%), polyurethane with polyvinyl chloride was used^(27)^; in two (16.7%), silicone gel^(15,28)^, and in one (8.3%), researchers compared hydrocolloid with polyvinyl chloride polyurethane foam^(18)^. In another study (8.3%), researchers investigated thin foam and hydrocolloid nasal protectors^(19)^ and, in one (8.3%)^(25)^, hydrocolloid was compared with silicone gel of different thicknesses. The time spent using interventions and monitoring ranged from less than two days^(28)^ up to four weeks^(17)^.

Most of the studies included in this review were carried out with premature NBs, the majority of which consisted of babies with a gestational age (GA) of 32 weeks or less. Birth weight ranged from 825.8 to 2,531.48 grams.

Chronologically, Chart 1 summarizes the characteristics of the studies that represented the *corpus* of this review.

**Chart 1:** Characteristics of the primary studies included in the systematic review – Santa Maria, RS, Brazil, 2024.

Author/year of publication/sample	Design of study	Intervention group (IG) (n)	Control group (CG) (n)	Respiratory device	Summary of main results
Günlemez et al., 2010^(15)^ n = 179 premature newborns	RCT	Silicone gel sheet (Epi-Derm Silicon Gel Sheeting, 1.8 mm thick, Biodermis) (n = 92)	No intervention (n = 87)	CPAP with nasal prongs	I: IG: 4.3% (n = 4) CG: 14.9% (n = 13) (OR: 3.43; 95% CI, 1.1–10.1; p < 0.05)
Xie, 2014^(23)^ n = 65 premature newborns prematuros	RCT	Hydrocolloid (1.8 mm thick, 90029T, 3M Company) (n = 33)	Paraffin oil (n = 32)	CPAP with nasal prongs	I: IG: 6.0% (n = 2) CG: 21.8% (n = 7) p = 0.01
Collins et al., 2014^(16)^ n = 132 premature newborns	RCT	HHHFNC group, all used Sticky Whiskers^®^ dressing (hydrocolloid) (n = 67)	Nasal CPAP group: Sticky Whiskers^®^ and Cannulaide^®^ (hydrocolloid) dressing (n = 65)	CPAP through binasal prongs and HHHFNC	Absence of data on I The mean nasal trauma score for infants in the HHHFNC**** group was 2.8 (SD 5.7), compared with 11.7 (SD 10.4) in the nasal CPAP** group (p < 0.001) There was no difference in mean trauma score between newborns on nasal CPAP who received Sticky Whiskers^®^ (14.4; SD 12.5) or Cannulaide^®^ (9.5; SD 7.3), p = 0.06
Morris et al., 2015^(17)^ n = 53 premature newborns	Cohort	Com-feel Plus™ Hydrocolloid Dressing (Coloplast Corp.) (n = 26)	Without barrier dressing (n = 27)	HHHFNC	Absence of data on I There was no statistical difference in nostril skin condition between groups (Wald *χ* ^2^ = 1.79 df = 1, p = 0.18) or over time (Wald *χ* ^2^ = 2.7 df = 3, p = 0.45)
Badr et al., 2016^(27)^ n = 101 premature newborns	Cohort	*NeoSeal* ^®^: Polyurethane foam dressing with polyvinyl chloride (PVC), foam, and pressure-sensitive adhesive (n = 51)	Without dressing (n = 50)	CPAP via nasal cannulas and intermittent positive airway pressure ventilation	I: Group without dressing: 24% (n = 12) Group *NeoSeal®*: 5.88% (n = 3) (OR = 4.08; 95% CI, 1.22–9.59; p = 0.01)
Imbulana et al., 2018^(24)^ n = 108 premature newborns	RCT	NeoGuard hydrocolloid dressing (Readmed Inc, Jiangsu Province, China; Australian Register of Therapeutic Goods Identifier 160458 Class 1) (n = 53)	Without dressing (n = 55)	Binasal prongs (CPAP and nasal intermittent positive pressure ventilation)	I: IG: 34.0% (n = 18) CG: 56.4% (n = 31) (p = 0.02)
Ribeiro et al., 2019^(28)^ n = 11 newborns	Case-control	Mölnlycke Health Care’s Mepiform® 0.3 mm thick self-adhesive silicone gel dressing. (n = 11)		NIV with short binasal prongs	I: 8 (72.7%)
Celda et al., 2020^(18)^ n = 13 newborns	Cohort	Group A: polyurethane foam dressing *NeoSeal®*, with polyvinyl chloride (PVC), foam and pressure-sensitive adhesive (n = 7)	Group B: Comfeel Plus extra-thin transparent hydrocolloid dressing (n = 6)	NIV (HFO), (CPAP/BiPAP) Interfaces: RAM cannula; nasal rods; nasal mask, nasal goggles	I: Group A: 40% (n = 2) Group B: 60% (n = 3) (*χ* ^2^ = 0.627, *p* = 0.429)
Ribeiro et al., 2020^(25)^ n = 33 premature newborns	RCT	Group A: transparent hydrocolloid (Comfeel Plus, Coloplast) (n = 11) Group B: thick silicone gel sheeting (Epi-Derm Silicone Gel Sheeting, Biodermis, 0.9 mm) (n = 11) Group C: thin adherent silicone (Mepiform, Molnlycke Health Care) (n = 11)	Not applicable	NIV with short binasal prongs	I: Group A: 36.36% (n = 4) Group B: 81.81% (n = 9) Group C: 72.72% (n = 8) (p = 0.06)
Yang et al., 2021^(20)^ n = 106 premature newborns	RCT	Hydrocolloid (n = 53)	U-shaped nasal plug, the type of material was not reported (n = 53)	Nasal CPAP	I: IG: 15% (n = 8) CG: 52.8% (n = 28) (p < 0.05)
Rezaei et al., 2021^(26)^ n = 80 premature newborns	RCT	Hydrocolloid dressing (Neo-Guard; ConLett) (n = 40)	Routine care (massage of the nasal septum and nostrils with 5% sodium chloride eye ointment) (n = 40)	CPAP with nasal prongs	I: IG: 37.5% (n = 15) CG: 92.5% (n = 37) (p < 0.001)
Nguyen et al., 2021^(19)^ n = 113 premature newborns	Cohort	Hydrocolloid dressing (n = 71)	1 mm thin foam nose protectors. Type of material not reported (n = 42)	Nasal cannula for NIV	Prevalence of nasal pressure ulcer Hydrocolloid group: 2.8% (n = 2) Fine foam group: 23.8% (n = 10) (OR = 0.09, 95% CI, 0.02 – 0.45) (p = 0.001)

Notes: RCT – randomized clinical trial; CPAP – continuous positive airway pressure; I – incidence of nasal pressure injury; HHHFNC – heated humidified high-flow nasal cannulae; NIV – non-invasive ventilation; HFO – high-flow oxygen therapy; BiPAP – bilevel positive airway pressure.

### Methodological Quality of Studies

The tools proposed by the JBI Collaboration were used for the methodological evaluation of the studies. It should be noted that none of them has a scoring system for the overall evaluation of the research, but a greater number of “yes” answers is indicative of better methodological quality.

Thus, through the tool JBI Critical Appraisal Checklist for Randomized Controlled Trial^(9)^ (n = 7) (Chart 2), of the 13 questions that make up the checklist, in three surveys^(16,24,26)^, eleven questions received the answer “yes”; in two^(15,20)^, nine questions and, in two studies^(23,25)^, eight questions. The question about blinding those who administered the treatment (in this case, applied the dressings) received the answer “no” in all studies. In all studies, the answers were “yes” to questions about the similarity between the intervention and control groups; blinding of participants to the intervention; administration of the intervention; follow-up; measurement of outcomes; and the use of appropriate statistical analysis.

**Chart 2:** JBI Critical Appraisal Checklist for Randomized Controlled Trial – Santa Maria, RS, Brazil, 2024.

Reference	Question												
	1	2	3	4	5	6	7	8	9	10	11	12	13
Günlemez et al., 2010^(15)^	N	N	Y	Y	N	Y	Y	Y	Y	Y	Y	Y	N
Xie, 2014^(23)^	N	N	Y	Y	N	N	Y	Y	Y	Y	Y	Y	N
Collins et al., 2014^(16)^	Y	Y	Y	Y	N	N	Y	Y	Y	Y	Y	Y	Y
Imbulana et al., 2018^(24)^	Y	Y	Y	Y	N	Y	Y	Y	NC	Y	Y	Y	Y
Ribeiro et al., 2020^(25)^	N	N	Y	Y	N	N	Y	Y	Y	Y	Y	Y	N
Yang et al., 2021^(20)^	Y	N	Y	Y	N	N	Y	Y	Y	Y	Y	Y	N
Rezaei et al.*,* 2021^(26)^	Y	Y	Y	Y	N	N	Y	Y	Y	Y	Y	Y	Y
**Percentage (%)**	57	43	100	100	0	28	100	100	85	100	100	100	43
Q1 – Was true randomization used to allocate participants to treatment groups? Q2 – Was the group allocation hidden? Q3 – Were the treatment groups similar at baseline? Q4 – Were participants blinded to treatment allocation? Q5 – Were those administering the treatment blinded to treatment allocation? Q6 – Were outcome assessors blinded to treatment allocation? Q7 – Were the treatment groups treated identically except for the intervention of interest? Q8 – Was follow-up complete and, if not, were differences between groups at follow-up adequately described and analyzed? Q9 – Were participants analyzed in the groups to which they were randomized? Q10 – Were the outcomes measured in the same way for the treatment groups? Q11 – Were the results measured reliably? Q12 – Was appropriate statistical analysis used? Q13 – Was the study design appropriate and were any deviations from standard RCT design taken into account in the conduct and analysis?													

Legend: Y = yes; N = no; NC = not clear. Source: authors; JBI^(9)^.

When applying the tool JBI Critical Appraisal Checklist for Cohort Studies^(9)^ (n = 4), of the 10 questions that make up the checklist, with answer options of “yes”, “no”, “not clear” and “not applicable”, the survey^(17)^ received a “no” response to four items about confounding factors and strategies to address them, about complete follow-up and strategies to address incomplete follow-up; the study^(27)^ received the answer “no” only to the question about approach strategies for incomplete follow-up; the survey^(18)^ received a “no” answer to questions about follow-up time and strategies to address incomplete follow-up; the study^(19)^ received the answer “no” to the questions about follow-up time, description of reasons for losses to follow-up, and strategies to address incomplete follow-up.

The questions directed at the recruitment of groups in relation to exposure, to valid and reliable measurements of exposures, to analysis of participants, whether they were free of the outcomes of interest at the beginning of the study, and to the statistical method used received a “yes” answer in the four studies. The question about strategies to address incomplete follow-up was not addressed in any of the included studies^(17, 18, 19,27)^.

The tool Critical Appraisal Checklist for Case-Control Studies^(9)^ (n = 1) contains 10 questions. The only case-control study included^(28)^ received a “no” response to three items related to the identification and management of confounding factors and the statistical analysis used. The remaining seven items, which address comparison between groups, adequate combination of cases and controls, identification criteria, measurement of exposure, standardized and reliable assessment of outcomes and period of exposure, received a “yes” answer.

### Network Meta-Analysis

The network meta-analysis for the outcome of nasal PU occurrence consisted of 571 premature newborns, the data were extracted from six randomized clinical trials^(15,20,23, 24, 25, 26)^. A study^(16)^ was not included because, despite meeting the review’s eligibility criteria, data on the occurrence of the event in absolute or relative frequency were not presented, hindering its inclusion in the quantitative models of this meta-analysis of dichotomous data (data were requested, but there was no response from the authors).

**Figure 2:**
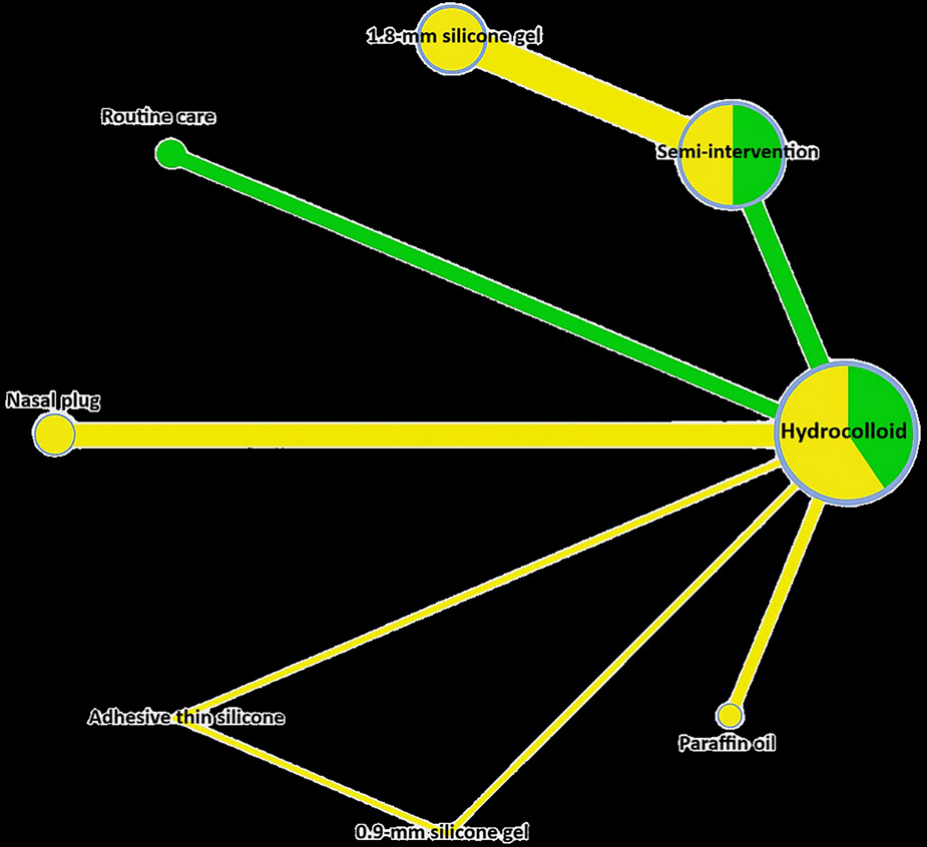
Geometry of the network of possible interventions to conduct the network meta-analysis obtained in the software Webapp Confidence in Network Meta-Analysis.

The structure of the interventions that allowed the network meta-analysis was presented graphically in Figure 2, a network of clinical trials comparing interventions for the prevention of nasal PU in newborns using respiratory medical devices. The edges width is proportional to the number of patients randomized in each comparison. The edges and nodes colors refer to the risk of bias, as follows: low, in green; moderate, in yellow. Furthermore, the circles represent the interventions and the straight lines are the direct comparisons. The greater the thickness, the greater the number of studies performing a given comparison.

In Table 1, direct and indirect comparisons of results on the effectiveness of prophylactic dressings for the occurrence of nasal PU in premature newborns using respiratory medical devices were presented. These results demonstrated that there was a significant difference between the interventions.

**Table 1:** League table of direct and indirect comparisons of results on the effectiveness of prophylactic dressings for the occurrence of nasal pressure injury in premature newborns using respiratory medical devices, in different groups – Santa Maria, RS, Brazil, 2024.

Routine Care	1.09 (0.43, 2.76)	**5.10 (1.48, 17. 61)**	**2.46 (1.63, 3.71)**	0.68 (0.14, 3.12)	0.70 (0.31, 1.56)	1.48 (0.81, 2.71)	1.23 (0.47, 3.20)
	0.9 mm Silicone Gel	**4.65 (1.11, 19.53)**	2.25 (0.98, 5.15)	0.62 (0.11, 3.44)	0.64 (0.21, 1.88)	1.35 (0.53, 3.47)	1.12 (0.71, 1.77)
		1.8 mm Silicone Gel	0.48 (0.15, 1.55)	**0.13 (0.02, 0.89)**	**0.13 (0.03, 0.53)**	**0.29 (0.09, 0.85)**	0.24 (0.05, 1.03)
			Hydrocolloid	0.27 (0.62, 1.23)	**0.28 (0.14, 0.56)**	**0.60 (0.38, 0.93)**	0.50 (0.21, 1.18)
				Paraffin Oil	1.03 (0.19, 5.34)	2.17 (0.45, 10.33)	1.80 (0.32,10.12)
					U-shaped nasal plug	2.10 (0.93, 4.77)	1.75 (0.58, 5.26)
						No Intervention	0.83 (0.31, 2.18)
							Thin Adhesive Silicone

Notes: Routine care, paraffin oil, nasal plug, thin adherent silicone, and no intervention are common comparators. Comparisons between interventions should be interpreted from left to right and top to bottom. For pairwise meta-analysis (upper right corner), RR (Relative Risk) was used, with its respective confidence intervals (CI). Source: authors; CINeMA, 2024.

It was found that routine care increases the risk of nasal PU occurrence, compared to 1.8 mm silicone gel (RR 5.10; 95% CI = 1.48, 17.61) and hydrocolloid (RR 2.46; 95% CI = 1.63, 3.71), as well as 0.9 mm silicone gel compared to 1.8 mm (RR 4.65; 95% CI = 1.11, 19.53). It was also observed that the use of 1.8 mm silicone reduces the risk of nasal PU, compared to paraffin oil (RR 0.13; 95% CI = 0.02, 0.89), nasal plug (RR 0.13; 95% CI = 0.03, 0.53) and not intervening (RR 0.29;95% CI = 0.09, 0.85), as well as the use of hydrocolloid reduces the risk of injury when compared to the plug (RR 0.28; 95% CI = 0.14, 0.56) and not intervening (RR 0.60; 95% CI = 0.38, 0.93).

### Certainty of Evidence

The CINeMA assessment indicated a “low” certainty of evidence for the outcome of nasal PU in newborns using a respiratory device, considering the interventions and comparators evaluated.

## DISCUSSION

The results of the review demonstrated the benefits of using prophylactic dressings to prevent the occurrence of nasal PU in premature newborns requiring ventilatory support, indicating different ways to intervene. Although studies with methodological differences were included, it was possible to construct a robust synthesis of evidence that can contribute to improving clinical practice.

Although unplanned, all studies included in the network meta-analysis were conducted with a population of preterm newborns. This is because respiratory failure related to lung immaturity is one of the most common immediate morbidities among premature infants, many of whom develop long-term complications^(29)^.

In the studies evaluated, there was a predominance of newborns who required CPAP and NIV. The use of NIV and nasal CPAP for respiratory support of premature NBs has been associated with a modest reduction in the incidence of lung damage, preventing progression to respiratory distress syndrome, especially if initiated prophylactically, before atelectasis manifests^(30)^.

The network meta-analysis indicated that routine care and use of 0.9 mm silicone gel do not contribute to reducing the occurrence of nasal PU in premature newborns. On the other hand, this outcome is prevented when using 1.8 mm silicone or hydrocolloid.

In a study, whose authors used an integrated experimental-computational approach in an adult individual, the biomechanical protection performance was compared between three foam-based dressings, one of which was silicone, and a hydrocolloid dressing to protect facial skin under a CPAP mask. In the nasal area, the difference in the calculation of the protective efficacy index was 86% for silicone foam compared to 60% for hydrocolloid^(4)^.

From this perspective, the prevention of nasal PU in NBs requiring respiratory support is a multifactorial challenge. Although the use of prophylactic dressings plays an important role in preventing this type of injury, and even if it proves effective in reducing this outcome, other factors must be carefully considered. The duration of ventilatory therapy, the type of device used and its interfaces are directly related to the etiology of this tissue damage^(18.26)^. Furthermore, characteristics of the NB, such as birth weight and gestational age, are significant determinants in increasing the risk of this condition^(19)^.

The emergence of PU increases hospital costs due to the management of secondary complications. In a systematic review, the authors evaluated the incidence, prevalence, attributable length of hospital stay, and cost of hospital-acquired PU in pediatrics, including NBs, with the results indicating a prevalence of 27.0% (95% CI: 22.1% – 33.1%) and an incidence of 9.8% (95% CI: 2.9% – 19.8%). For the general pediatric population, length of stay ranged from 0.9 to 14.1 days, and attributable cost ranged from $894.69 to $98,730.24 (United States dollars; 2020 dollar exchange value) per patient with nosocomial PU^(31)^. Therefore, preventive strategies, such as the use of these protective coverings, can generate long-term savings by reducing the incidence of injuries and hospitalization costs, in addition to minimizing avoidable damage, aligning with the principles of patient safety and good practices in neonatology.

When applying the methodological quality assessment instruments for randomized clinical trials, it was possible to analyze that in some studies data on randomization and allocation were not presented^(15,23,25)^, as well as those who administered the dressings^(15,20,23, 24, 25, 26)^ and who assessed the outcome were not blinded^(16,20,23,25,26)^. However, in these studies, blinding professionals and evaluators is unfeasible due to the very nature of the intervention and requires an application, such as dressings, that the professional needs to be familiar with. This implies bias related to selection and allocation, administration of the intervention/exposure, and evaluation, detection and measurement of the outcome, which may compromise the internal validity of these studies^(32)^.

In two observational studies included in the review, the results demonstrated that the intervention (prophylactic dressing) led to a reduction in nasal PU in NBs using ventilatory therapy equipment, indicating the benefit of its application^(19,27)^. However, in the other three studies, the results did not demonstrate benefits of the intervention, as they did not show improvements in the prevention of this condition^(17,18,28)^. These variations and inconsistencies between the results of observational studies may be related to possible biases identified during the application of methodological quality assessment instruments, as some did not find confounding factors nor showed the strategies to deal with them. Furthermore, some did not describe complete follow-up or reasons for losses and did not present strategies for dealing with incomplete follow-up^(17, 18, 19,27)^.

The certainty of evidence classified as “low” indicates limited effect confidence and that it may be influenced by further investigations, an aspect that may be attributed to the presence of selection bias in some included studies, related to the lack of adequate randomization and lack of allocation concealment; also, due to the presence of differences in interventions within the studies included, referring to the type of material and lack of standardization in the comparators, in addition to limitations in the samples and number of events in both the intervention group and the comparators.

As an implication for research, to strengthen future recommendations aimed at implementing Evidence-Based Practice in clinical practice, we highlight the need to conduct studies with robust methodological quality, with representative samples, rigorous bias control, standardization of investigated interventions, including consistent criteria for evaluating outcomes. Furthermore, factors including the duration and type of respiratory device and its interfaces, as well as the characteristics of the NB, such as birth weight and gestational age, should be carefully considered in future studies.

In this context, the selection of effective skin protective barriers impacts clinical outcomes and the costs associated with healthcare. Therefore, understanding mechanisms such as the dressing’s ability to reduce stiffness gradients between equipment materials and the skin is relevant. The dressing should facilitate an increase in the contact area between the contours of the device and the skin surface, enabling the redistribution of loads. These properties are essential for reducing stress concentrations in the skin and underlying tissues, contributing to the prevention of this tissue damage^(33)^.

As an implication for practice, the results of the network meta-analysis support the indication of using prophylactic hydrocolloid and 1.8 mm silicone gel dressings as effective interventions to prevent nasal PU in premature newborns, between the surface of the nostrils and the respiratory equipment interface. For this purpose, the dressings can be cut to the shape of the nasal base, creating two holes adapted to the anatomy of the nose and nostrils, allowing for proper positioning of the device and promoting skin protection^(20,23,25)^.

Thus, the evidence generated in this review can support healthcare professionals’ decision-making, suggesting that the inclusion of prophylactic dressings as part of the neonatal skin care routine can improve patient safety and the quality of care provided to this vulnerable population.

### Study Limitations

While meta-analyses represent the best evidence, the data from this review need to be interpreted considering the particularities of the primary studies included. Moreover, data missing from some of the included studies, the heterogeneity of interventions tested, and the way nasal PU occurrence was assessed limited further comparisons.

## CONCLUSION

This review identified evidence favorable to the use of prophylactic dressings in the prevention of nasal PU in premature newborns using non-invasive respiratory support devices. Hydrocolloid and 1.8 mm thick silicone gel dressings stood out as effective interventions in mitigating this problem. However, the methodological quality of the studies included in the network meta-analysis resulted in a level of certainty of evidence classified as low, which constitutes limitations to the generalization of the findings and demands caution in their incorporation into clinical practice.

Nevertheless, the results of this systematic review provide support for neonatal care practice, suggesting the judicious use of prophylactic dressings as an adjuvant preventive strategy in NICUs. However, the need for new RCTs with consistent methodological designs is highlighted to consolidate scientific evidence on the effectiveness and safety of these interventions. Thus, this study contributes to the existing body of knowledge, as it outlines gaps that guide future research in the area.

## Data Availability

The dataset that supports the findings of this study is not publicly available.
