# Supplementary material for: Prophylactic dressings for preventing nasal pressure ulcer in premature newborns: an effectiveness review
Source: Rev Esc Enferm USP. 2025 Nov 28;59:e20250099. doi: 10.1590/1980-220X-REEUSP-2025-0099en (PMC12670641; doi:10.1590/1980-220X-REEUSP-2025-0099en)
Supplement: Translation

# Translation: Curativos profiláticos para prevenção de lesão por pressão nasal em neonatos prematuros: uma revisão de efetividade

**DOI:** 10.1590/1980-220X-REEUSP-2025-0099pt

**Authors:** Cibelle Mello Viero, Paulo Jorge Pereira Alves, Oclaris Lopes Munhoz, Thaynan Silveira Cabral, Cristina Maria Galvão, Valdecir Zavarese da Costa, Thaís Dresch Eberhardt, Matheus Silvelo Franco, Suzinara Beatriz Soares de Lima

**Affiliations:** 1 Universidade Federal de Santa Maria, Centro de Ciências da Saúde, Programa de Pós-Graduação de Enfermagem, Santa Maria, RS, Brasil.; 2 Universidade Católica Portuguesa, Faculdade de Ciências da Saúde e Enfermagem, Porto, Portugal.; 3 Universidade Federal de Santa Maria, Departamento de Ciências da Saúde, Palmeira das Missões, RS, Brasil.; 4 Universidade de São Paulo, Escola de Enfermagem, Ribeirão Preto, SP, Brasil.; 5 Universidade Federal de Santa Maria, Centro de Ciências da Saúde, Departamento de Enfermagem, Santa Maria, RS, Brasil.; 6 Universidade de Passo Fundo, Passo Fundo, RS, Brasil.

**Keywords:** Úlcera por Pressão, Recém-nascido, Unidades de Terapia Intensiva Neonatal, Revisão Sistemática, Prática Clínica Baseada em Evidências

## Abstract

**Objetivo:**

Avaliar a efetividade de curativos profiláticos para prevenção de lesão por pressão nasal em neonatos em uso de dispositivo médico respiratório.

**Método:**

Revisão sistemática de efetividade, segundo a metodologia JBI. Incluíram-se estudos com neonatos em uso de dispositivo médico respiratório, que receberam intervenção de curativos profiláticos. Realizadas síntese descritiva e metanálise em rede. Registro PROSPERO CRD42024516296.

**Resultados:**

Foram incluídos 12 estudos, com um total de 1.001 neonatos. O uso de silicone 1,8 mm reduz o risco de ocorrência de lesão por pressão nasal, comparado a óleo de parafina (RR 0,13; IC95% = 0,02, 0,89), a plug nasal (RR 0,13; IC 95% = 0,03, 0,53) e a não intervir (RR 0,29; IC 95% = 0,09, 0,85), assim como utilizar hidrocoloide reduz o risco de lesão quando comparado ao plug (RR 0,28; IC 95% = 0,14, 0,56) e a não intervir (RR 0,60; IC 95% = 0,38, 0,93).

**Conclusão:**

Curativos profiláticos, sobretudo gel de silicone 1,8 mm e hidrocoloide, são efetivos na prevenção de lesões por pressão nasais em recém-nascidos prematuros que utilizam dispositivos respiratórios.

## INTRODUÇÃO

Destaca-se que a estrutura da pele de prematuros é qualitativa e quantitativamente distinta da pele de neonatos a termo e lactentes. Além disso, os mecanismos de dano tissular nessa população apresentam características únicas^(1)^. Nesse contexto, recém-nascido (RN) admitido em unidade de terapia intensiva neonatal (UTIN) tem risco para desenvolver danos cutâneos, principalmente lesão por pressão (LP), que consiste em pro­blema que merece a atenção dos profissionais de saúde. As LP relacionadas a dispositivos médicos estão entre as mais frequentes, atingindo 73,5%, sobretudo associadas à pressão positiva contínua nas vias aéreas (CPAP)^(2)^.

O uso prolongado de máscaras de ventilação, como CPAP, expõe os tecidos moles faciais a um risco aumentado de rupturas devido às deformações contínuas da pele e dos tecidos subjacentes na interface de contato com esses equipamentos^(3,4)^.

Apesar do uso de curativos profiláticos ser recomendado em diretrizes clínicas internacionais e ser considerado padrão de cuidado em alguns contextos clínicos, ainda não existe a uniformização e consenso entre os pesquisadores para avaliar a função preventiva desses materiais^(5)^. Entretanto, sabe-se que o principal requisito deste tipo de cobertura é a redução das cargas teciduais localizadas e sustentadas nos tecidos moles, entre o dispositivo e a pele^(6)^.

A aplicação de determinadas barreiras protetoras cutâneas, como hidrocoloides e espumas, na interface entre a pele e o sistema de suporte respiratório, como a máscara de terapia ventilatória, pode minimizar o risco de LP ao redistribuir as forças de contato localizadas e dissipar as tensões superficiais e internas nos tecidos. Dessa forma, os profissionais de saúde devem adotar abordagens objetivas, padronizadas e baseadas em evidências na tomada de decisão na prática clínica para selecionar os produtos mais efetivos na prevenção deste agravo^(4)^.

Diante do exposto, uma busca preliminar foi realizada nas seguintes fontes de informações: *International Prospective Register of Systematic Reviews* (PROSPERO), MEDLINE, *Cochrane Database of Systematic Reviews* e Colaboração JBI, sendo que revisões sistemáticas atuais ou em andamento não foram identificadas sobre a efetividade de curativos profiláticos para a prevenção de LP nasal em neonatos. Encontrou-se um protocolo com o objetivo de avaliar as evidências quanto aos cuidados preventivos de LP por CPAP nasal em neonatos prematuros^(7)^ e outra revisão que investigou os benefícios do curativo hidrocoloide na prevenção de dano tissular nasal em bebês em uso de CPAP, com limitação de ano de publicação e idioma^(8)^. Portanto, é pertinente buscar, avaliar e sintetizar as evidências sobre a temática.

Ao reunir e analisar criticamente as evidências disponíveis, este estudo pretende contribuir para a qualificação do cuidado de enfermagem neonatal, fornecendo subsídios científicos para o desenvolvimento de protocolos assistenciais mais efetivos e baseados em evidências. Dessa forma, busca-se fortalecer a segurança e a qualidade do cuidado prestado aos neonatos, reduzindo a ocorrência de LP nasais e seus impactos na saúde dessa população vulnerável. Assim, o objetivo do estudo foi avaliar a efetividade de curativos profiláticos para prevenção de LP nasal em neonatos em uso de dispositivo médico respiratório, comparada a grupo controle, placebo ou cuidado habitual.

## MÉTODO

### Tipo de Estudo, Protocolo e Registro

Revisão sistemática de efetividade com metanálise em rede desenvolvida segundo a metodologia JBI^(9)^ e relatada em conformidade com o *Preferred Reporting Items for Systematic Reviews and Meta-Analyses* (PRISMA)^(10)^. Protocolo de revisão registrado na plataforma PROSPERO (CRD42024516296).

A pergunta da revisão foi estruturada com base no acrônimo PICO, no qual P (população) = neonatos em uso de dispositivo médico respiratório; I (intervenção) = curativos profiláticos; C (comparador) = grupo controle, placebo ou cuidado habitual; e O (desfecho) = ocorrência de lesão por pressão nasal. Logo, questiona-se: qual é a efetividade de curativos profiláticos para prevenção de LP nasais em neonatos em uso de dispositivos médicos respiratórios, em comparação ao grupo controle, placebo ou aos cuidados habituais?

### Critérios de Elegibilidade

Foram incluídos estudos experimentais e observacionais com a participação de RN^(11)^ hospitalizados em uso de dispositivos médicos respiratórios (equipamentos ou parte deles que estivessem em contato direto com a pele para administração de terapia ventilatória)^(12)^, que receberam intervenção com curativos profiláticos com geis ou espumas, como silicone, hidrocoloide, poliuretano e polivinila, aplicados na pele antes da ocorrêcia de dano tissular^(13)^. Ainda, investigações que compararam a intervenção com cuidados não padronizados ou sem intervenção a placebo, óleos, ou cuidados habituais foram incluídas.

Foram excluídas as pesquisas que envolviam RN com LP nasal ou cujo foco fosse a investigação do uso de curativos para tratamento desse tipo de lesão. Não foi definida delimitação de idioma ou recorte temporal e as produções duplicadas foram analisadas somente uma vez.

### Fontes de Informação e Amostragem

As buscas dos estudos primários foram realizadas em abril de 2024, nas seguintes fontes de informação: Biblioteca Virtual em Saúde (BVS), *Medical Literature Analysis and Retrieval System Online (MEDLINE),* via Pubmed*, Scientific Electronic Library Online (SciELo) e Cochrane Library, EMBASE e Scopus (Elsevier), Web of Science (Clarivate), Academic Search Premier, Cumulative Index to Nursing and Allied Health Literature (CINAHL) with Full Text (EBSCO), SCIENCE e La Reference.* Também foi conduzida uma busca complementar no site *Google Scholar* utilizando os títulos e autores dos estudos incluídos na revisão.

Estratégias de buscas avançadas foram realizadas, considerando as especificidades de cada fonte de informação. Assim, foram empregados os vocabulários, Descritores em Ciências da Saúde (DeCS), sinônimos, *Medical Subject Headings (MESH), entry terms* e *CINAHL headings*, os quais foram combinados com os operadores booleanos *AND e OR*. Uma bibliotecária, com expertise na elaboração de estratégias de busca na condução de métodos de síntese de conhecimento, realizou a revisão das estratégias construídas para as diferentes fontes de informação.

Na sequência, a estratégia de busca delimitada para a base de dados MEDLINE foi apresentada, a saber: (((“Pressure Ulcer”[mh] OR Bed Sore* OR Bedsore* OR Decubitus Ulcer* OR Pressure Sore* OR Pressure Ulcer* OR decubital ulcer* OR decubitus ulceration OR pressure injur* OR pressure sore OR decubitus OR Nasal injuries OR “Nasal Septum”[mh] OR Nasal Septum* Nasal OR Nose[mh] OR Nose* OR Friction[mh] OR Friction OR Pressure[mh] OR Pressure*) AND (“Infant, Newborn”[mh] OR Newborn Infant* OR Newborn* OR Neonate* OR Infant[mh] OR Infant* OR “Child, Preschool”[mh] OR Preschool* OR Child[mh] OR Children OR “full term infant” OR neonatus OR “newly born baby” OR “newly born child” OR “newly born infant” OR Pediatric* OR “Infant, Premature”[mh] OR Preterm* OR Premature* OR “Neonatal Prematurity” OR prematurita* OR Neonatology[mh] OR Neonatolog*)) AND (“Continuous Positive Airway Pressure”[mh] OR Airway Pressure Release Ventilation OR APRV Ventilation Mode OR Bilevel Continuous Positive Airway Pressure OR Bilevel Positive Airway Pressure OR BiPAP OR Bilevel Positive Airway Pressure OR Biphasic Positive Airway Pressure OR Biphasic Continuous Positive Airway Pressure OR Biphasic Positive Airway Pressure OR CPAP Ventilation OR CPAP OR Nasal Continuous Positive Airway Pressure OR nCPAP Ventilation OR “Noninvasive Ventilation”[mh] OR Non Invasive Ventilation* OR Non-Invasive Ventilation* OR “Noninvasive Ventilations” OR Device* OR Medical Device* OR “Medical device-related pressure injuries” OR “Positive-Pressure Respiration”[mh] OR Positive End Expiratory Pressure OR Positive End-Expiratory Pressure* OR Positive Pressure Respiration OR Positive Pressure Ventilation OR Positive-Pressure Respirations OR Positive-Pressure Ventilation* OR “Respiration, Artificial”[mh] OR Artificial Respiration* OR Mechanical Ventilation* OR NCPAP OR “high flow nasal cannula”)) AND (Bandages[mh] OR Bandage OR Dressing* OR “Bandages, Hydrocolloid”[mh] OR Duoderm OR Hydrocolloid Bandage* OR Hydrocolloid Dressing* OR Hydrogel Bandage* OR “wound care device” OR “wound care product” OR “wound therapy device” OR “bandages and dressings” OR “Comfeel Plus” OR hydrocolloid OR duoderm OR Exuderm OR granuflex OR Tegasorb OR Varihesive OR Prophylactic dressing* OR Preventive dressing* OR Protective dressing* OR Preventive wound management OR Silicone dressings OR Silicone bandages OR Silicone wound dressings OR Silicone wound covers Polyvinyl dressings OR Polyvinyl bandages OR Polyvinyl wound dressings OR Polyvinyl wound covers OR “prevention & control” OR “preventive therapy”). A partir dessa estratégia, as adaptações para as demais fontes de informação foram realizadas. Devido ao número limitado de páginas do manuscrito, não foi possível incluir integralmente as estratégias de busca das diferentes fontes de informação; no entanto, elas estão disponíveis no registro do protocolo da revisão sistemática na plataforma PROSPERO.

### Triagem e Seleção dos Estudos

Todas as citações identificadas foram agrupadas e carregadas no *software EndNote* (*Clarivate,* PA, EUA), as duplicadas removidas e importadas com detalhes de citação para o *software Rayyan: Systematic Review Management* ^(14)^, para realização da triagem e seleção.

A seleção dos estudos primários foi conduzida por dois revisores, de forma cega e independente. O processo teve início com a leitura dos títulos e resumos, seguida pela análise completa dos textos selecionados, de acordo com os critérios de elegibilidade. Em seguida, os revisores compararam seus resultados para identificar divergências, resolvendo-as por consenso. A participação de um terceiro revisor estava prevista para casos de discordância; no entanto, não houve necessidade.

### Avaliação da Qualidade Metodológica

Foram utilizados os instrumentos padronizados pela Colaboração JBI para ensaio clínico randomizado, estudos de coorte e caso-controle. Essas ferramentas são listas de verificação que possuem, respectivamente, treze, onze e dez itens (perguntas), com opções de respostas de sim, não, incerto/não claro ou não aplicável^(9)^.

Os estudos elegíveis foram avaliados criticamente por dois revisores independentes e as divergências resolvidas com auxílio de um terceiro revisor. Todos os estudos, independentemente de sua qualidade metodológica, passaram por extração e síntese de dados.

### Extração dos Dados

Foram extraídos os seguintes dados: características do estudo (autores, ano, idioma, país, delineamento do estudo, cenário, tipo de instrumento/coleta de dados) e da amostra (número de neonatos, idade gestacional e peso ao nascer, sexo, tipo de dispositivo médico respiratório), tipo de intervenção entre os grupos, tempo de utilização da intervenção, principais resultados e conclusões relacionados a LP nasal (incidência/prevalência das lesões nos diferentes grupos – diferenças estatísticas, classificação das lesões em estágios). Os dados coletados foram organizados em tabelas no Google Docs e posteriormente importados para o *Microsoft Word* ^®^ e os desfechos inseridos no programa *Microsoft Excel* ^®^, por um revisor, com verificação cruzada independente do outro.

Ressalta-se que, em seis estudos incluídos^(15-20)^ não constavam todas as informações necessárias para extração de dados e, na ocasião, entrou-se em contato com os autores via e-mail e rede social *ResearchGate,* com o tempo de espera de 45 dias; apenas um^(15)^ disponibilizou os resultados solicitados.

### Síntese das Evidências

Todos os estudos incluídos foram considerados para a síntese de evidências. Para os estudos observacionais, elaborou-se uma síntese descritiva, com uso de frequências absoluta (n) e relativa (%).

Para os estudos experimentais, realizou-se uma metanálise em rede (*Network Meta-Analysis* – *NMA*), de modelo de efeitos aleatórios, com comparações diretas e indiretas dos estudos experimentais^(21)^. Também se considerou o princípio da transitividade, que requer que as intervenções concorrentes de uma revisão sistemática sejam randomizáveis em conjunto^(22)^ e, como medida de efeito, o Risco Relativo (RR). A metanálise foi conduzida utilizando o CINeMA (*Confidence in network meta-analysis*), vinculado ao software R^®^, sendo empregado especialmente para calcular a contribuição de estudos nos efeitos relativos^(21,22)^. A tabela de liga foi extraída do *software* CINeMA.

Embora a revisão tenha sido planejada para incluir neonatos em geral, todos os estudos elegíveis para a NMA avaliaram exclusivamente neonatos prematuros. Dessa forma, a síntese estatística foi conduzida como uma análise de subgrupo, restrita a essa população.

### Avaliação da Certeza da Evidência

Utilizou-se o *software Webapp* do CINeMA para a avaliação da certeza da evidência, a qual abrange seis domínios: risco de viés nos estudos analisados, viés de relato/publicação, evidência indireta, imprecisão, heterogeneidade e incoerência. Para cada domínio, são atribuídos três níveis de preocupação: “sem preocupações”, “algumas preocupações” e “grandes preocupações”, exceto para o domínio “viés de relato”, que é classificado como “baixo risco”, “algumas preocupações” e “alto risco”. A certeza da evidência pode ser categorizada como “muito baixa”, “baixa”, “moderada” ou “alta”, e nesta revisão foi atribuída para o desfecho ocorrência de LP nasal^(21,22)^.

## RESULTADOS

### Inclusão dos Estudos

Identificou-se um total de 809 registros nas fontes de informações, dos quais 184 eram duplicados, restando 625 publicações para leitura de títulos e resumos. Destas, 604 foram excluídas por não atenderem aos critérios de seleção. Na etapa de elegibilidade, 21 publicações foram lidas na íntegra e 12 permaneceram para a síntese de evidências. Um fluxograma detalhado da seleção dos estudos foi apresentado na Figura 1.

**Figura 1:** Fluxograma de seleção dos estudos – Preferred Reporting Items for Systematic Review and Meta-Analyses (PRISMA)^(10)^.

### Caracterização dos Estudos Incluídos

Em relação ao delineamento do estudo, sete (53%) constituíram-se em ensaio clínico randomizado (ECR)^(15,16,20,23–26)^, quatro (33%) em estudo de coorte^(17–19,27)^ e um (8,3%) estudo caso-controle^(28)^.

Dos 12 estudos incluídos, um (8,3%) foi originário da Turquia^(15)^, dois (16,7%) da China^(20,23)^, dois (16,7%) da Austrália^(16,24)^, um (8,3%) dos Estados Unidos da América^(17)^, dois (16,7%) do Brasil^(25,28)^, um (8,3%) da Espanha^(18)^, um (8,3%) do Líbano^(27)^, um (8,3%) do Irã^(26)^ e um (8,3%) do Vietnã^(19)^. A amostra total foi de 1.001 RN (soma de todas as amostras dos estudos incluídos), o período de publicação variou de 2010 a 2021, 11 (91,7%) foram publicados no idioma inglês e um (8,3%) no espanhol^(18)^.

Em relação ao tipo de dispositivo médico respiratório e suas interfaces: quatro (33,3%) estudos utilizaram somente o CPAP nasal^(20)^ com prongas nasais^(15,23,26)^, um (8,3%) utilizou cânula nasal para ventilação não invasiva (VNI)^(19)^, dois (16,7%) VNI por meio de prongas binasais curtas^(25,28)^, um (8,3%) o CPAP por meio de prongas binasais e as cânulas nasais de alto fluxo aquecidas e umidificadas, do inglês, *heated humidified high-flow nasal cannulae* (HHHFNC)^(16)^, um (8,3%) a cânula nasal via CPAP e/ou ventilação com pressão positiva intermitente nas vias aéreas nasais^(27)^; um (8,3%), prongas binasais via CPAP ou ventilação com pressão positiva intermitente nasal^(24)^; um (8,3%), somente prongas para cânula nasal umidificada e aquecida de alto fluxo^(17)^; um (8,3%) utilizou VNI via oxigenoterapia de alto fluxo (OAF) e/ou CPAP/*Bilevel Positive Airway Pressure* (BiPAP), pressão positiva em dois níveis nas vias aéreas, por meio de cânula RAM®, hastes nasais, máscara nasal, e óculos nasais^(18)^.

No que tange ao material interventivo testado, em seis pesquisas (50%), os autores aplicaram o curativo de hidrocoloide^(16,17,20,23,24,26)^; em uma (8,3%) foi utilizado o de poliuretano com cloreto de polivinila^(27)^; em duas (16,7%), o silicone gel^(15,28)^ e, em uma (8,3%), os pesquisadores compararam o hidrocoloide com espuma de poliuretano com cloreto de polivinila^(18)^. Em outro estudo (8,3%), os estudiosos investigaram protetores nasais de espuma fina e hidrocoloide^(19)^ e, em um (8,3%)^(25)^ comparou-se hidrocoloide com silicone gel de diferentes espessuras. Já o tempo de utilização das intervenções e acompanhamento variou de menos de dois dias^(28)^ até quatro semanas^(17)^.

A maior parte dos estudos incluídos nesta revisão foi realizada com RN prematuros, sendo a maioria composta por bebês com idade gestacional (IG) de 32 semanas ou menos. O peso ao nascer variou de 825,8 a 2.531,48 gramas.

De forma cronológica, o Quadro 1 sintetiza as características dos estudos que representaram o *corpus* desta revisão.

**Quadro 1:** Características dos estudos primários incluídos na revisão sistemática – Santa Maria, RS, Brasil, 2024.

Autor/ano de publicação/amostra	Tipo de estudo	Grupo intervenção (GI) (n)	Grupo controle (GC) (n)	Dispositivo respiratório	Síntese dos principais resultados
Günlemez et al., 2010(15) n = 179 neonatos prematuros	ECR	Folha de gel de silicone (Epi-Derm Silicon Gel Sheeting, 1,8 mm de espessura, Biodermis) (n = 92)	Sem intervenção (n = 87)	CPAP com prongas nasais	I: GI: 4,3% (n = 4) GC: 14,9% (n = 13) (OR: 3,43; IC 95%, 1,1–10,1; p < 0,05)
Xie, 2014^(23)^ n = 65 neonatos prematuros	ECR	Hidrocoloide (1,8 mm de espessura, 90029T, 3M Company) (n = 33)	Óleo de parafina (n = 32)	CPAP com prongas nasais	I: GI: 6,0% (n = 2) GC: 21,8% (n = 7) p = 0,01
Collins et al., 2014^(16)^ n = 132 neonatos prematuros	ECR	Grupo HHHFNC, todos utilizaram curativo Sticky Whiskers®. (hidrocoloide) (n = 67)	Grupo CPAP nasal: curativo Sticky Whiskers® e Cannulaide® (hidrocoloide) (n = 65)	CPAP por meio de prongas binasais e HHHFNC	Ausência de dados sobre I A pontuação média de trauma nasal para os lactentes do grupo HHHFNC**** foi de 2,8 (DP 5,7), comparada a 11,7 (DP 10,4) no grupo CPAP** nasal (p < 0,001) Não houve diferença na pontuação média de trauma entre os neonatos em CPAP nasal que receberam Sticky Whiskers® (14,4; DP 12,5) ou Cannulaide® (9,5; DP 7,3), p = 0,06
Morris et al., 2015^(17)^ n = 53 neonatos prematuros	Coorte	Curativo hidrocoloide Com-feel Plus™ (Coloplast Corp.) (n = 26)	Sem curativo barreira (n = 27)	HHHFNC	Ausência de dados sobre I Não houve diferença estatística na condição da pele das narinas entre os grupos (Wald *χ*2 = 1.79df = 1, p = 0,18) ou ao longo do tempo (Wald *χ*2 = 2.7df = 3, p = 0,45)
Badr et al., 2016^(27)^ n = 101 neonatos prematuros	Coorte	NeoSeal®: Curativo de espuma de poliuretano com cloreto de polivinila (PVC), espuma e adesivo sensível à pressão (n = 51)	Sem curativo (n = 55)	CPAP por cânulas nasais e ventilação com pressão positiva intermitente nas vias aéreas nasais	I: Grupo sem curativo: 24% (n = 12) Grupo NeoSeal®: 5,88% (n = 3) (OR = 4,08; 95% CI, 1,22–9,59; p = 0,01)
Imbulana et al., 2018^(24)^ n = 108 neonatos prematuros	ECR	Curativo de hidrocoloide NeoGuard (Readmed Inc, Jiangsu Province, China; Australian Register of Therapeutic Goods Identifier 160458 Class 1) (n = 53)	Sem curativo (n = 55)	Prongas binasais (CPAP e ventilação nasal com pressão positiva intermitente)	I: GI: 34,0% (n = 18) GC: 56,4% (n = 31) (p = 0,02)
Ribeiro et al., 2019^(28)^ n = 11 neonatos	Caso-controle	Curativo autoadesivo de silicone gel da Mölnlycke Health Care’s Mepiform® 0.3 mm de espessura. (n = 11)	VNI com prongas binasais curtas	I: 8 (72,7%)
Celda et al., 2020^(18)^ n = 13 neonatos	Coorte	Grupo A: curativo de espuma de poliuretano NeoSeal®, com cloreto de polivinila (PVC), espuma e adesivo sensível à pressão (n = 7)	Grupo B: curativo hidrocoloide extrafino Comfeel Plus transparente (n = 6)	VNI (OAF), (CPAP/BiPAP) Interfaces: cânula RAM; hastes nasais; máscara nasal, óculos nasais	I: Grupo A: 40% (n = 2) Grupo B: 60% (n = 3) (*χ*2 = 0,627, p = 0,429)
Ribeiro et al., 2020^(25)^ n = 33 neonatos prematuros	ECR	Grupo A: hidrocoloide transparente (Comfeel Plus, Coloplast) (n = 11) Grupo B: fita de gel silicone espesso (Epi-Derm Silicone Gel Sheeting, Biodermis, 0,9 mm) (n = 11) Grupo C: silicone fino aderente (Mepiform, Molnlycke Health Care) (n = 11)	Não se aplica	VNI com prongas binasais curtas	I: Grupo A: 36,36% (n = 4) Grupo B: 81,81% (n = 9) Grupo C: 72,72% (n = 8) (p = 0,06)
Yang et al., 2021^(20)^ n = 106 neonatos prematuros	ECR	Hidrocoloide (n = 53)	Plug nasal em formato de U, o tipo de material não foi informado (n = 53)	CPAP nasal	I: GI: 15% (n = 8) GC: 52,8% (n = 28) (p < 0,05)
Rezaei et al., 2021^(26)^ n = 80 neonatos prematuros	ECR	Curativo de hidrocoloide (Neo-Guard; ConLett) (n = 40)	Cuidados de rotina (massagem do septo nasal e narinas com pomada ocular de cloreto de sódio a 5%) (n = 40)	CPAP com prongas nasais	I: GI: 37,5% (n = 15) GC: 92,5% (n = 37) (p < 0,001)
Nguyen et al., 2021^(19)^ n = 113 neonatos prematuros	Coorte	Curativo hidrocoloide (n = 71)	Protetores nasais de espuma fina de 1 mm. Tipo de material não informado (n = 42)	Cânula nasal para VNI	Prevalência de lesão por pressão nasal Grupo hidrocoloide: 2,8% (n = 2) Grupo espuma fina: 23,8% (n = 10) (OR = 0,09, IC 95%, 0,02–0,45) (p = 0,001)

Notas: ECR – ensaio clínico randomizado; CPAP – *continuous positive airway pressure* (pressão positiva contínua nasal de vias aéreas); I – incidência de lesão por pressão nasal; HHHFNC – *heated humidified high-flow nasal* cannulae (cânula nasal de alto fluxo aquecida e umidificada); VNI – ventilação não invasiva; OAF – oxigenoterapia de alto fluxo; BiPAP – *bilevel positive airway pressure* (pressão positiva em dois níveis nas vias aéreas).

### Qualidade Metodológica dos Estudos

As ferramentas propostas pela Colaboração JBI foram empregadas para a avaliação metodológica dos estudos. Salienta-se que nenhuma delas tem um sistema de escore para avaliação geral da pesquisa, mas um número maior de respostas “sim” é indicativo de melhor qualidade metodológica.

Assim, por meio da ferramenta *JBI Critical Appraisal Checklist for Randomized Controlled Trial* ^(9)^ (n = 7) (Quadro 2), das 13 perguntas que compõem o checklist, em três pesquisas^(16,24,26)^, onze perguntas receberam a resposta “sim”; em duas^(15,20)^, nove perguntas e, em dois estudos^(23,25)^, oito perguntas. A pergunta sobre o cegamento dos que administraram o tratamento (no caso, aplicaram os curativos) recebeu a resposta “não” em todos os estudos. Em todas as pesquisas, as respostas foram “sim” para as perguntas sobre a semelhança entre os grupos intervenção e controle; cegamento dos participantes em relação à intervenção; na administração da intervenção; no acompanhamento; na medição dos desfechos e, no uso de análise estatística apropriada.

**Quadro 2:** JBI Critical Appraisal Checklist for Randomized Controlled Trial – Santa Maria, RS, Brasil, 2024.

Referência	Questão												
	1	2	3	4	5	6	7	8	9	10	11	12	13
Günlemez et al., 2010^(15)^	N	N	S	S	N	S	S	S	S	S	S	S	N
Xie, 2014^(23)^	N	N	S	S	N	N	S	S	S	S	S	S	N
Collins et al., 2014^(16)^	S	S	S	S	N	N	S	S	S	S	S	S	S
Imbulana et al., 2018^(24)^	S	S	S	S	N	S	S	S	NC	S	S	S	S
Ribeiro et al., 2020^(25)^	N	N	S	S	N	N	S	S	S	S	S	S	N
Yang et al., 2021^(20)^	S	N	S	S	N	N	S	S	S	S	S	S	N
Rezaei et al.*,* 2021^(26)^	S	S	S	S	N	N	S	S	S	S	S	S	S
**Percentual (%)**	57	43	100	100	0	28	100	100	85	100	100	100	43
Q1 – A verdadeira randomização foi utilizada para alocação de participantes nos grupos de tratamento? Q2 – A alocação dos grupos foi ocultada? Q3 – Os grupos de tratamento eram semelhantes na linha de base? Q4 – Os participantes estavam cegados para a alocação do tratamento? Q5 – Aqueles que administraram o tratamento estavam cegos para a alocação do tratamento? Q6 – Os avaliadores de resultados estavam cegos para a alocação do tratamento? Q7 – Os grupos de tratamento foram tratados de forma idêntica, exceto pela intervenção de interesse? Q8 – O acompanhamento foi completo e, em caso negativo, as diferenças entre os grupos em relação ao acompanhamento foram adequadamente descritas e analisadas? Q9 – Os participantes foram analisados nos grupos para os quais foram randomizados? Q10 – Os resultados foram medidos da mesma forma para os grupos de tratamento? Q11 – Os resultados foram medidos de maneira confiável? Q12 – Foi utilizada uma análise estatística apropriada? Q13 – O desenho do estudo foi apropriado e quaisquer desvios do desenho padrão de ECR foram levados em consideração na condução e análise?													

Legenda: S = sim; N = não; NC = não claro. Fonte: autores; JBI^(9)^.

Ao aplicar a ferramenta *JBI Critical Appraisal Checklist for Cohort Studies* ^(9)^ (n = 4), das 10 perguntas que compõem o checklist, com opções de respostas de “sim”, “não”, “não claro” e “não se aplica”, a pesquisa^(17)^ recebeu resposta “não” para quatro itens sobre os fatores de confusão e estratégias para lidar com eles, sobre o acompanhamento completo e estratégias para abordar o acompanhamento incompleto; o estudo^(27)^ recebeu a resposta “não” somente para a pergunta sobre estratégias de abordagem para o acompanhamento incompleto; a pesquisa^(18)^ recebeu resposta “não” para as questões sobre o tempo de acompanhamento e estratégias para abordar o acompanhamento incompleto; o estudo^(19)^ recebeu a resposta “não” para as perguntas sobre o tempo de acompanhamento, descrição de motivos de perdas de acompanhamento e estratégias para abordar o acompanhamento incompleto.

As questões direcionadas para o recrutamento dos grupos em relação à exposição, para aferições das exposições de forma válida e confiável, para análise dos participantes, se estavam livres dos desfechos de interesse no início do estudo e para o método estatístico empregado receberam resposta “sim” nos quatro estudos. A pergunta sobre estratégias para abordar o acompanhamento incompleto não foi contemplada em nenhum dos estudos incluídos^(17–19,27)^.

A ferramenta *Critical Appraisal Checklist for Case-Control Studies* ^(9)^ (n = 1) contém 10 questões. O único estudo de caso-controle incluído^(28)^ recebeu resposta “não” em três itens relacionados à identificação e manejo de fatores de confusão e à análise estatística utilizada. Os demais sete itens, que abordam comparação entre grupos, combinação adequada de casos e controles, critérios de identificação, medição da exposição, avaliação padronizada e confiável dos desfechos e período de exposição, receberam resposta “sim”.

### Metanálise em Rede

A metanálise em rede para o desfecho ocorrência de LP nasal foi composta por 571 RN prematuros, os dados foram extraídos de seis ensaios clínicos randomizados^(15,20,23–26)^. Um estudo^(16)^ não foi incluído, pois apesar de atender aos critérios de elegibilidade da revisão, os dados sobre a ocorrência do evento em frequência absoluta ou relativa não foram apresentados, impossibilitando a inclusão nos modelos quantitativos desta metanálise de dados dicotômicos (os dados foram solicitados, mas não houve retorno dos autores).

A estrutura das intervenções que permitiram a metanálise em rede foi apresentada graficamente na Figura 2, uma rede de ensaios clínicos comparando as intervenções para a prevenção de LP nasal em neonatos que utilizaram dispositivos médicos respiratórios. A largura das bordas é proporcional ao número de pacientes randomizados em cada comparação. As cores das bordas e nós referem-se ao risco de viés, sendo que: baixo, em verde; moderado, em amarelo. Ainda, os círculos representam as intervenções e as linhas retas são as comparações diretas. Quanto maior a espessura, maior o número de estudos que realizaram determinada comparação.

**Figura 2:** Geometria da rede de intervenções possíveis de conduzir a metanálise em rede obtida no *software Webapp Confidence in Network Meta-Analysis*.

Na Tabela 1, as comparações diretas e indiretas de resultados sobre a efetividade de curativos profiláticos para a ocorrência de LP nasal em neonatos prematuros em uso de dispositivo médico respiratório foram apresentadas. Tais resultados demonstraram que houve diferença significativa entre as intervenções.

**Tabela 1:** Tabela de liga (*league table*) de comparações diretas e indiretas de resultados sobre a efetividade de curativos profiláticos para a ocorrência de lesão por pressão nasal em neonatos prematuros em uso de dispositivo médico respiratório, em diferentes grupos – Santa Maria, RS, Brasil, 2024.

Cuidados de Rotina	1,09 (0,43, 2,76)	**5,10 (1,48, 17, 61)**	**2,46 (1,63, 3,71)**	0,68 (0,14, 3,12)	0,70 (0,31, 1,56)	1,48 (0,81, 2,71)	1,23 (0,47, 3,20)
	Gel de Silicone 0,9 mm	**4,65 (1,11, 19,53)**	2,25 (0,98, 5,15)	0,62 (0,11, 3,44)	0,64 (0,21, 1,88)	1,35 (0,53, 3,47)	1,12 (0,71, 1,77)
		Gel de Silicone 1,8 mm	0,48 (0,15, 1,55)	**0,13 (0,02, 0,89)**	**0,13 (0,03, 0,53)**	**0,29 (0,09, 0,85)**	0,24 (0,05, 1,03)
			Hidrocoloide	0,27 (0,62, 1,23)	**0,28 (0,14, 0,56)**	**0,60 (0,38, 0,93)**	0,50 (0,21, 1,18)
				Óleo de Parafina	1,03 (0,19, 5,34)	2,17 (0,45, 10,33)	1,80 (0,32,10,12)
					Plug Nasal U	2,10 (0,93, 4,77)	1,75 (0,58, 5,26)
						Sem Intervenção	0,83 (0,31, 2,18)
							Silicone Fino Aderente

Notas: Cuidados de rotina, óleo de parafina, plug nasal, silicone fino aderente e ausência de intervenção são comparadores comuns. As comparações entre as intervenções devem ser interpretadas da esquerda para a direita e de cima para baixo. Para metanálise de pares (canto superior direito) utilizou-se o RR (Risco Relativo), com seus respectivos intervalos de confiança (IC). Fonte: autores; CINeMA, 2024.

Constatou-se que os cuidados de rotina aumentam o risco de ocorrência de LP nasal, comparado a gel de silicone 1,8 mm (RR 5,10; IC 95% = 1,48, 17,61) e a hidrocoloide (RR 2,46; IC 95% = 1,63, 3,71), assim como o gel de silicone 0,9 mm comparado ao de 1,8 mm (RR 4,65; IC 95% = 1,11, 19,53). Também se verificou que o uso de silicone 1,8 mm reduz o risco de ocorrência de LP nasal, comparado a óleo de parafina (RR 0,13; IC 95% = 0,02, 0,89), a plug nasal (RR 0,13; IC 95% = 0,03, 0,53) e a não intervir (RR 0,29; IC 95% = 0,09, 0,85), assim como utilizar hidrocoloide reduz o risco de lesão quando comparado ao plug (RR 0,28; IC 95% = 0,14, 0,56) e a não intervir (RR 0,60; IC 95% = 0,38, 0,93).

### Certeza da Evidência

A avaliação pelo CINeMA apontou uma certeza de evidência “baixa” para o desfecho ocorrência de LP nasal em neonatos em uso de dispositivo respiratório, considerando as intervenções e comparadores avaliados.

## DISCUSSÃO

Os resultados da revisão demonstraram os benefícios de utilizar curativos profiláticos para prevenir a ocorrência de LP nasal em neonatos prematuros que necessitam de suporte ventilatório, indicando diferentes formas de intervir. Por mais que tenham sido incluídos estudos com diferenças metodológicas, possibilitou-se construir uma síntese de evidências robusta que pode contribuir para a melhoria da prática clínica.

Embora não planejado, todos os estudos incluídos na metanálise em rede foram conduzidos com população de neonatos prematuros. Isso se deve à insuficiência respiratória relacionada à imaturidade pulmonar ser uma das morbidades imediatas mais comuns entre prematuros, muitos dos quais desenvolvem complicações de longo prazo^(29)^.

Nas pesquisas avaliadas, houve predomínio de neonatos que necessitaram de CPAP e VNI. O uso da VNI e do CPAP nasal para suporte respiratório de RN prematuro tem sido associado a uma redução modesta na incidência de danos pulmonares, prevenindo a progressão para a síndrome do desconforto respiratório, principalmente se iniciado profilaticamente, antes que a atelectasia se manifeste^(30)^.

A metanálise em rede indicou que os cuidados de rotina e uso de gel de silicone 0,9 mm não contribuem para a diminuição da ocorrência de LP nasal em neonatos prematuros. Por outro lado, tal desfecho é prevenido quando se utiliza silicone 1,8 mm ou hidrocoloide.

Em um estudo, cujos autores utilizaram uma abordagem experimental-computacional integrada de um indivíduo adulto, o desempenho de proteção biomecânica foi comparado de três curativos à base de espuma, sendo um desses com silicone, com o de hidrocoloide para proteger a pele facial sob uma máscara de CPAP. Na área nasal, a diferença do cálculo do índice de eficácia protetora foi de 86% para o de espuma com silicone comparado a 60% para o hidrocoloide^(4)^.

Nesta perspectiva, a prevenção de LP nasal em RN que necessita de suporte respiratório é um desafio multifatorial. Embora o uso de curativo profilático desempenhe papel importante na prevenção deste tipo de lesão, e mesmo se mostrando efetivo para a redução deste desfecho, outros fatores devem ser cuidadosamente considerados. A duração da terapia ventilatória, o tipo de dispositivo utilizado e suas interfaces estão diretamente relacionados à etiologia desse dano tissular^(18,26)^. Outrossim, características do RN, como o peso ao nascer e a idade gestacional, são determinantes significativos no aumento do risco para esse agravo^(19)^.

O surgimento de LP aumenta os custos hospitalares devido ao manejo de complicações secundárias. Em revisão sistemática, os autores avaliaram a incidência, prevalência, tempo de internação atribuível e custo de LP adquiridas no hospital em pediatria, incluindo neonatos, sendo que os resultados indicaram prevalência de 27,0% (IC 95%: 22,1% – 33,1%) e incidência de 9,8% (IC 95%: 2,9% – 19,8%). Para a população pediátrica em geral, o tempo de internação variou de 0,9 a 14,1 dias, e o custo atribuível variou de $894,69 a $98.730,24 (dólares dos Estados Unidos da América; valor de um dólar em 2020) por paciente com LP nosocomiais^(31)^. Destarte, estratégias preventivas, como o uso dessas coberturas protetoras, podem gerar economia em longo prazo ao reduzir a incidência de lesões e os custos de hospitalização, além de minimizar danos evitáveis, alinhando-se aos princípios da segurança do paciente e de boas práticas em neonatologia.

Ao aplicar os instrumentos de avaliação de qualidade metodológica para ensaio clínico randomizado, foi possível analisar que em alguns estudos não foram apresentados dados sobre a randomização e alocação^(15,23,25)^, assim como os que administravam os curativos^(15,20,23–26)^ e que avaliavam o desfecho não estavam cegados^(16,20,23,25,26)^. No entanto, nesses estudos, o cegamento dos profissionais e avaliadores é inviável devido à própria natureza da intervenção e necessita de uma aplicação que o profissional precisa conhecer, a exemplo dos curativos. Isso implica em viés relacionado à seleção e alocação, à administração da intervenção/exposição e à avaliação, detecção e medição do resultado, o que pode comprometer a validade interna dessas pesquisas^(32)^.

Em dois estudos observacionais incluídos na revisão, os resultados demonstraram que a intervenção (curativo profilático) acarretou redução de LP nasal em RN em uso de equipamentos de terapia ventilatória, indicando o benefício de sua aplicação^(19,27)^. Contudo, nos outros três estudos, os resultados não demonstraram benefícios da intervenção, pois não evidenciaram melhorias na prevenção desse agravo^(17,18,28)^. Essas variações e inconsistências entre os resultados dos estudos observacionais podem estar relacionadas a possíveis vieses identificados durante a aplicação dos instrumentos de avaliação da qualidade metodológica, pois alguns não apresentaram a identificação de fatores de confusão, nem a declaração de estratégias para lidar com eles. Além disso, alguns não descreveram o acompanhamento completo ou os motivos das perdas e não apresentaram estratégias para lidar com o acompanhamento incompleto^(17–19,27)^.

A certeza da evidência classificada como “baixa” indica uma confiança de efeito limitada e que pode ser influenciada por novas investigações, aspecto que pode ser atribuído à presença de viés de seleção em alguns estudos incluídos, relacionado à falta de randomização adequada e falta de ocultação da alocação; também pela presença de diferenças nas intervenções entre os estudos incluídos, referentes ao tipo de material e falta de padronização nos comparadores, além de limitação nas amostras e número de eventos tanto no grupo de intervenção quanto nos comparadores.

Como implicação para pesquisa, para fortalecer recomendações futuras tendo como propósito a implementação da Prática Baseada em Evidências na prática clínica, salienta-se a necessidade de condução de estudos com qualidade metodológica robusta, com amostras representativas, controle rigoroso de viés, padronização de intervenções investigadas, incluindo critérios consistentes para a avaliação de desfechos. Ainda, fatores incluindo a duração e o tipo de dispositivo respiratório e suas interfaces, bem como as características do RN, como peso ao nascer e idade gestacional, devem ser cuidadosamente considerados em estudos futuros.

Nesse contexto, a seleção de barreiras protetoras cutâneas efetivas exerce impacto sobre os desfechos clínicos e sobre os custos associados ao cuidado em saúde. Assim, entender mecanismos como a capacidade do curativo de reduzir os gradientes de rigidez entre os materiais dos equipamentos e a pele é relevante. O curativo deve favorecer o aumento da área de contato entre os contornos do dispositivo e a superfície cutânea, possibilitando a redistribuição das cargas. Essas propriedades são fundamentais para a redução das concentrações de estresse na pele e nos tecidos subjacentes, contribuindo para a prevenção desse dano tissular^(33)^.

Como implicação para prática, os resultados da metanálise em rede oferecem suporte para a indicação da utilização de curativos profiláticos de hidrocoloide e silicone gel 1,8 mm como intervenções efetivas para prevenir LP nasal em neonatos prematuros, entre a superfície das narinas e a interface do equipamento respiratório. Para essa finalidade, os curativos podem ser recortados no formato da base nasal com a confecção de dois orifícios adaptados à anatomia do nariz e das narinas, permitindo adequado posicionamento do dispositivo e promovendo a proteção da pele^(20,23,25)^.

Assim, as evidências geradas na condução desta revisão podem subsidiar a tomada de decisão dos profissionais da saúde, sugerindo que a inclusão de curativos profiláticos como parte da rotina de cuidado à pele neonatal pode melhorar a segurança do paciente e qualidade dos cuidados prestados a essa população vulnerável.

### Limitações do Estudo

Ao passo em que metanálises configuram-se como a melhor evidência, os dados da presente revisão precisam ser interpretados considerando as particularidades dos estudos primários incluídos. Também, os dados não encontrados em alguns dos estudos incluídos, a heterogeneidade de intervenções testadas e a forma de avaliação de ocorrência de LP nasal limitaram outras comparações.

## CONCLUSÃO

A presente revisão identificou evidências favoráveis ao uso de curativos profiláticos na prevenção de LP nasal em RN prematuros que utilizam dispositivos de suporte respiratório não invasivo. As coberturas de hidrocoloide e as de gel de silicone com espessura de 1,8 mm destacaram-se como intervenções efetivas na mitigação desse agravo. Todavia, a qualidade metodológica dos estudos incluídos na metanálise em rede resultou em um nível de certeza da evidência classificado como baixo, o que configura limitações à generalização dos achados e demanda cautela na sua incorporação à prática clínica.

Ainda assim, os resultados desta revisão sistemática fornecem subsídios para a prática assistencial neonatal, sugerindo a utilização criteriosa de curativos profiláticos como estratégia preventiva adjuvante nas UTIN. Ressalta-se, entretanto, a necessidade de novos ECR, com desenhos metodológicos consistentes a fim de consolidar a evidência científica sobre a efetividade e segurança dessas intervenções. Assim, este estudo contribui para o corpo de conhecimento existente, à medida que delineia lacunas que orientam futuras investigações na área.

## Data Availability

Conjunto de dados que dá suporte aos resultados deste estudo não está disponível publicamente.

# Translation: Vendajes profilácticos para prevenir úlceras por presión nasal en neonatos prematuros: una revisión de su eficacia

**DOI:** 10.1590/1980-220X-REEUSP-2025-0099es

**Authors:** Cibelle Mello Viero, Paulo Jorge Pereira Alves, Oclaris Lopes Munhoz, Thaynan Silveira Cabral, Cristina Maria Galvão, Valdecir Zavarese da Costa, Thaís Dresch Eberhardt, Matheus Silvelo Franco, Suzinara Beatriz Soares de Lima

**Affiliations:** 1 Universidade Federal de Santa Maria, Centro de Ciências da Saúde, Programa de Pós-Graduação de Enfermagem, Santa Maria, RS, Brasil.; 2 Universidade Católica Portuguesa, Faculdade de Ciências da Saúde e Enfermagem, Porto, Portugal.; 3 Universidade Federal de Santa Maria, Departamento de Ciências da Saúde, Palmeira das Missões, RS, Brasil.; 4 Universidade de São Paulo, Escola de Enfermagem, Ribeirão Preto, SP, Brasil.; 5 Universidade Federal de Santa Maria, Centro de Ciências da Saúde, Departamento de Enfermagem, Santa Maria, RS, Brasil.; 6 Universidade de Passo Fundo, Passo Fundo, RS, Brasil.

**Keywords:** Úlcera por Presión, Recién Nacido, Unidades de Cuidado Intensivo Neonatal, Revisión Sistemática, Práctica Clínica Basada en la Evidencia

## Abstract

**Objetivo:**

Evaluar la eficacia de los vendajes profilácticos para prevenir úlceras por presión nasal en neonatos que utilizan dispositivos médicos respiratorios.

**Método:**

Revisión sistemática de efectividad, según la metodología JBI. Se incluyeron estudios que incluyeron neonatos que utilizaron dispositivos médicos respiratorios y que recibieron una intervención de vendaje profiláctico. Se realizó síntesis descriptiva y metanálisis en red. Matrícula PROSPERO CRD42024516296.

**Resultados:**

Se incluyeron 12 estudios, con un total de 1.001 neonatos. El uso de silicona de 1,8 mm reduce el riesgo de úlcera por presión nasal, en comparación con el aceite de parafina (RR 0,13; IC 95% = 0,02, 0,89), tapón nasal (RR 0,13; IC 95% = 0,03, 0,53) y no intervenir (RR 0,29; IC 95% = 0,09, 0,85), así como el uso de hidrocoloide reduce el riesgo de úlcera en comparación con el tapón (RR 0,28; IC 95% = 0,14, 0,56) y no intervenir (RR 0,60; IC 95% = 0,38, 0,93).

**Conclusión:**

Los vendajes profilácticos, especialmente los de gel de silicona de 1,8 mm y los hidrocoloides, son eficaces para prevenir las lesiones por presión nasal en recién nacidos prematuros que utilizan dispositivos de respiración.

## INTRODUCCIÓN

Es necesario señalar en primer lugar que la estructura de la piel de los prematuros es cualitativa y cuantitativamente distinta de la piel de los recién nacidos a término y los lactantes. Además, los mecanismos de daño tisular en esta población presentan características únicas^(1)^. En este contexto, los recién nacidos (RN) ingresados en una unidad de terapia intensiva neonatal (UTIN) corren el riesgo de desarrollar daños cutáneos, principalmente lesiones por presión (LP), lo que constituye un problema que merece la atención de los profesionales de la salud. Las LP relacionadas con dispositivos médicos se encuentran entre las más frecuentes, alcanzando el 73,5%, sobre todo asociadas a la presión positiva continua en las vías respiratorias (CPAP)^(2)^.

El uso prolongado de máscaras de ventilación, como la CPAP, expone los tejidos blandos faciales a un mayor riesgo de roturas debido a las deformaciones continuas de la piel y los tejidos subyacentes en la interfaz de contacto con estos equipos^(3,4)^.

A pesar de que el uso de curativos profilácticos se recomienda en las directrices clínicas internacionales y se considera un estándar de atención en algunos contextos clínicos, todavía no existe uniformidad ni consenso entre los investigadores para evaluar la función preventiva de estos materiales^(5)^. Sin embargo, se sabe que el principal requisito de este tipo de cobertura es la reducción de las cargas tisulares localizadas y sostenidas en los tejidos blandos, entre el dispositivo y la piel^(6)^.

La aplicación de determinadas barreras protectoras cutáneas, como hidrocoloides y espumas, en la interfaz entre la piel y el sistema de soporte respiratorio, como la máscara de terapia ventilatoria, puede minimizar el riesgo de LP al redistribuir las fuerzas de contacto localizadas y disipar las tensiones superficiales e internas en los tejidos. Por lo tanto, los profesionales de la salud deben adoptar enfoques objetivos, estandarizados y basados en la evidencia en la toma de decisiones en la práctica clínica para seleccionar los productos más eficaces en la prevención de esta afección^(4)^.

En vista de lo anterior, se realizó una búsqueda preliminar en las siguientes fuentes de información: Registro Internacional Prospectivo de Revisiones Sistemáticas (PROSPERO), MEDLINE, Base de Datos Cochrane de Revisiones Sistemáticas y Colaboración JBI, sin que se identificaran revisiones sistemáticas actuales o en curso sobre la eficacia de los curativos profilácticos para la prevención de la LP nasal en recién nacidos. Se encontró un protocolo con el objetivo de evaluar las evidencias sobre los cuidados preventivos de la LP por CPAP nasal en neonatos prematuros^(7)^ y otra revisión que investigó los beneficios del apósito hidrocoloide en la prevención del daño tisular nasal en bebés que utilizan CPAP, con limitación de año de publicación e idioma^(8)^.

Por lo tanto, es pertinente buscar, evaluar y sintetizar las evidencias sobre el tema. Al reunir y analizar críticamente las evidencias disponibles, este estudio pretende contribuir a la calificación de la atención de enfermería neonatal, proporcionando subsidios científicos para el desarrollo de protocolos de atención más eficaces y basados en la evidencia.

De esta manera, se busca fortalecer la seguridad y la calidad de la atención prestada a los recién nacidos, reduciendo la incidencia de LP nasales y sus impactos en la salud de esta población vulnerable. Así, el objetivo del estudio fue evaluar la eficacia de los curativos profilácticos para la prevención de LP nasales en recién nacidos que utilizan dispositivos médicos respiratorios, en comparación con un grupo control, un placebo o la atención habitual.

## MÉTODO

### Tipo de Estudio, Protocolo y Registro

Revisión sistemática de la eficacia con metaanálisis en red desarrollada según la metodología JBI^(9)^ y reportada de acuerdo con los *Preferred Reporting Items for Systematic Reviews and Meta-Analyses* (PRISMA)^(10)^. Protocolo de revisión registrado en la plataforma PROSPERO (CRD42024516296).

La pregunta de la revisión se estructuró basándose en el acrónimo PICO, en el que P (población) = recién nacidos que utilizan dispositivos médicos respiratorios; I (intervención) = curativos profilácticos; C (comparador) = grupo control, placebo o atención habitual; y O (resultado) = aparición de lesiones por presión nasal. Por lo tanto, la pregunta es: ¿cuál es la eficacia de los curativos profilácticos para la prevención de las lesiones por presión nasal en neonatos que utilizan dispositivos médicos respiratorios, en comparación con el grupo control, el placebo o la atención habitual?

### Criterios de Elegibilidad

Se incluyeron estudios experimentales y observacionales con la participación de recién nacidos^(11)^ hospitalizados que utilizaban dispositivos médicos respiratorios (equipos o partes de ellos que estaban en contacto directo con la piel para la administración de terapia ventilatoria)^(12)^, que recibieron una intervención con curativos profilácticos con geles o espumas, como silicona, hidrocoloide, poliuretano y polivinilo, aplicados sobre la piel antes de que se produjera el daño tisular^(13)^. Además, se incluyeron investigaciones que compararon la intervención con cuidados no estandarizados o sin intervención con placebo, aceites, o cuidados habituales.

Se excluyeron las investigaciones que involucraban a recién nacidos con LP nasal o cuyo enfoque era la investigación del uso de curativos para el tratamiento de este tipo de lesión. No se definió ninguna delimitación de idioma o periodo de tiempo, y las producciones duplicadas se analizaron solo una vez.

### Fuentes de Información y Muestreo

Las búsquedas de los estudios primarios se realizaron en abril de 2024 en las siguientes fuentes de información: Biblioteca Virtual en Salud (BVS), *Medical Literature Analysis and Retrieval System Online (MEDLINE),* a través de Pubmed*, Scientific Electronic Library Online (SciELO) y Cochrane Library, EMBASE y Scopus (Elsevier), Web of Science (Clarivate), Academic Search Premier, Índice Acumulativo de Literatura de Enfermería y Salud Aliada (CINAHL) con texto completo (EBSCO), SCIENCE y La Reference.* También se realizó una búsqueda complementaria en el sitio web *Google Scholar* utilizando los títulos y autores de los estudios incluidos en la revisión.

Se llevaron a cabo estrategias de búsqueda avanzadas, teniendo en cuenta las especificidades de cada fuente de información. Así, se emplearon los vocabularios Descriptores en Ciencias de la Salud (DeCS), sinónimos, Medical Subject Headings (MESH), *entry terms* y CINAHL *headings*, que se combinaron con los operadores booleanos AND y OR. Una bibliotecaria, con experiencia en la elaboración de estrategias de búsqueda en la conducción de métodos de síntesis de conocimiento, realizó la revisión de las estrategias construidas para las diferentes fuentes de información.

A continuación, se presentó la estrategia de búsqueda delimitada para la base de datos MEDLINE, a saber: (((“Pressure Ulcer”[mh] OR Bed Sore* OR Bedsore* OR Decubitus Ulcer* OR Pressure Sore* OR Pressure Ulcer* OR decubital ulcer* OR decubitus ulceration OR pressure injur* OR pressure sore OR decubitus OR Nasal injuries OR “Nasal Septum”[mh] OR Nasal Septum* Nasal OR Nose[mh] OR Nose* OR Friction[mh] OR Friction OR Pressure[mh] OR Pressure*) AND (“Infant, Newborn”[mh] OR Newborn Infant* OR Newborn* OR Neonate* OR Infant[mh] OR Infant* OR “Child, Preschool”[mh] OR Preschool* OR Child[mh] OR Children OR “full term infant” OR neonatus OR “newly born baby” OR “newly born child” OR “newly born infant” OR Pediatric* OR “Infant, Premature”[mh] OR Preterm* OR Premature* OR “Neonatal Prematurity” OR prematurita* OR Neonatology[mh] OR Neonatolog*)) AND (“Continuous Positive Airway Pressure”[mh] OR Airway Pressure Release Ventilation OR APRV Ventilation Mode OR Bilevel Continuous Positive Airway Pressure OR Bilevel Positive Airway Pressure OR BiPAP OR Bilevel Positive Airway Pressure OR Biphasic Positive Airway Pressure OR Biphasic Continuous Positive Airway Pressure OR Biphasic Positive Airway Pressure OR CPAP Ventilation OR CPAP OR Nasal Continuous Positive Airway Pressure OR nCPAP Ventilation OR “Noninvasive Ventilation”[mh] OR Non Invasive Ventilation* OR Non-Invasive Ventilation* OR “Noninvasive Ventilations” OR Device* OR Medical Device* OR “Medical device-related pressure injuries” OR “Positive-Pressure Respiration”[mh] OR Positive End Expiratory Pressure OR Positive End-Expiratory Pressure* OR Positive Pressure Respiration OR Positive Pressure Ventilation OR Positive-Pressure Respirations OR Positive-Pressure Ventilation* OR “Respiration, Artificial”[mh] OR Artificial Respiration* OR Mechanical Ventilation* OR NCPAP OR “high flow nasal cannula”)) AND (Bandages[mh] OR Bandage OR Dressing* OR “Bandages, Hydrocolloid”[mh] OR Duoderm OR Hydrocolloid Bandage* OR Hydrocolloid Dressing* OR Hydrogel Bandage* OR “wound care device” OR “wound care product” OR “wound therapy device” OR “bandages and dressings” OR “Comfeel Plus” OR hydrocolloid OR duoderm OR Exuderm OR granuflex OR Tegasorb OR Varihesive OR Prophylactic dressing* OR Preventive dressing* OR Protective dressing* OR Preventive wound management OR Silicone dressings OR Silicone bandages OR Silicone wound dressings OR Silicone wound covers Polyvinyl dressings OR Polyvinyl bandages OR Polyvinyl wound dressings OR Polyvinyl wound covers OR “prevention & control” OR “preventive therapy”). A partir de esta estrategia, se realizaron las adaptaciones para las demás fuentes de información. Debido al número limitado de páginas del manuscrito, no fue posible incluir íntegramente las estrategias de búsqueda de las diferentes fuentes de información; sin embargo, estas están disponibles en el registro del protocolo de la revisión sistemática en la plataforma PROSPERO.

### Selección de los Estudios

Todas las citas identificadas se agruparon y se cargaron en el software EndNote (Clarivate, PA, EE. UU.), se eliminaron las duplicadas y se importaron con los detalles de la cita al software Rayyan: Systematic Review Management^(14)^, para realizar el cribado y la selección.

La selección de los estudios primarios fue realizada por dos revisores, de forma ciega e independiente. El proceso comenzó con la lectura de los títulos y resúmenes, seguida de un análisis completo de los textos seleccionados, de acuerdo con los criterios de elegibilidad. A continuación, los revisores compararon sus resultados para identificar divergencias, resolviéndolas por consenso. Se previó la participación de un tercer revisor en caso de desacuerdo; sin embargo, no fue necesario.

### Evaluación de la Calidad Metodológica

Se utilizaron los instrumentos estandarizados por la Colaboración JBI para ensayos clínicos aleatorios, estudios de cohortes y estudios de casos y controles. Estas herramientas son listas de verificación que contienen, respectivamente, trece, once y diez ítems (preguntas), con opciones de respuesta sí, no, incierto/no claro o no aplicable^(9)^.

Los estudios elegibles fueron evaluados críticamente por dos revisores independientes y las discrepancias se resolvieron con la ayuda de un tercer revisor. Todos los estudios, independientemente de su calidad metodológica, se sometieron a extracción y síntesis de datos.

### Extracción de Datos

Se extrajeron los siguientes datos: características del estudio (autores, año, idioma, país, diseño del estudio, escenario, tipo de instrumento/recopilación de datos) y de la muestra (número de recién nacidos, edad gestacional y peso al nacer, sexo, tipo de dispositivo médico respiratorio), tipo de intervención entre los grupos, tiempo de uso de la intervención, principales resultados y conclusiones relacionadas con la LP nasal (incidencia/prevalencia de las lesiones en los diferentes grupos: diferencias estadísticas, clasificación de las lesiones en etapas). Los datos recopilados se organizaron en tablas en Google Docs y posteriormente se importaron a Microsoft Word® y los resultados se introdujeron en el programa Microsoft Excel®, por un revisor, con verificación cruzada independiente del otro.

Cabe destacar que en seis de los estudios incluidos^(15-20)^ no figuraba toda la información necesaria para la extracción de datos y, en ese momento, se contactó a los autores por correo electrónico y a través de la red social *ResearchGate,* con un tiempo de espera de 45 días; solo uno^(15)^ proporcionó los resultados solicitados.

### Síntesis de la Evidencia

Todos los estudios incluidos se consideraron para la síntesis de la evidencia. Para los estudios observacionales, se elaboró una síntesis descriptiva, utilizando frecuencias absolutas (n) y relativas (%).

Para los estudios experimentales, se realizó un metaanálisis en red (*Network Meta-Analysis* – *NMA*), de modelo de efectos aleatorios, con comparaciones directas e indirectas de los estudios experimentales^(21)^. También se consideró el principio de transitividad, que requiere que las intervenciones concurrentes de una revisión sistemática sean aleatorizables en conjunto^(22)^ y, como medida de efecto, el riesgo relativo (RR). El metaanálisis se realizó utilizando CINeMA (Confidence in network meta-analysis), vinculado al software R^®^, que se empleó especialmente para calcular la contribución de los estudios en los efectos relativos^(21,22)^. La tabla de ligas se extrajo del software CINeMA.

Aunque la revisión se planificó para incluir a los recién nacidos en general, todos los estudios elegibles para el NMA evaluaron exclusivamente a los recién nacidos prematuros. Por lo tanto, la síntesis estadística se realizó como un análisis de subgrupo, restringido a esta población.

### Evaluación de la Certeza de la Evidencia

Se utilizó el software Webapp de CINeMA para evaluar la certeza de la evidencia, que abarca seis ámbitos: riesgo de sesgo en los estudios analizados, sesgo de notificación/publicación, evidencia indirecta, imprecisión, heterogeneidad e incoherencia. A cada dominio se le asignan tres niveles de preocupación: “sin preocupaciones”, “algunas preocupaciones” y “grandes preocupaciones”, excepto para el dominio “sesgo de informe”, que se clasifica como “bajo riesgo”, “algunas preocupaciones” y “alto riesgo”. La certeza de la evidencia se puede categorizar como “muy baja”, “baja”, “moderada” o “alta”, y en esta revisión se asignó al resultado de la ocurrencia de LP nasal^(21,22)^.

## RESULTADOS

### Inclusión de los Estudios

Se identificaron un total de 809 registros en las fuentes de información, de los cuales 184 eran duplicados, quedando 625 publicaciones para la lectura de títulos y resúmenes. De estas, 604 fueron excluidas por no cumplir los criterios de selección. En la etapa de elegibilidad, se leyeron 21 publicaciones en su totalidad y 12 quedaron para la síntesis de la evidencia. En la Figura 1 se presenta un diagrama de flujo detallado de la selección de los estudios.

**Figura 1:** Flujograma de selección de estudios – Preferred Reporting Items for Systematic Review and Meta-Analyses (PRISMA)^(10)^.

### Caracterización de los Estudios Incluidos

En cuanto al diseño del estudio, siete (53 %) fueron ensayos clínicos randomizados (ECR)^(15,16,20,23-26)^, cuatro (33 %) fueron estudios de cohortes^(17–19, 27)^ y uno (8,3 %) fue un estudio de casos y controles^(28)^.

De los 12 estudios incluidos, uno (8,3 %) procedía de Turquía^(15)^, dos (16,7 %) de China^(20,23)^, dos (16,7 %) de Australia^(16,24)^, uno (8,3 %) de los Estados Unidos de América^(17)^, dos (16,7 %) de Brasil^(25,28)^, uno (8,3 %) de España^(18)^, uno (8,3 %) del Líbano^(27)^, uno (8,3 %) de Irán^(26)^ y uno (8,3%) de Vietnam^(19)^. La muestra total fue de 1001 recién nacidos (suma de todas las muestras de los estudios incluidos), el período de publicación varió de 2010 a 2021, 11 (91,7%) se publicaron en inglés y uno (8,3%) en español^(18)^.

En cuanto al tipo de dispositivo médico respiratorio y sus interfaces: cuatro (33,3%) estudios utilizaron solo CPAP nasal^(20)^ con cánulas nasales^(15,23,26)^, uno (8,3%) utilizó una cánula nasal para ventilación no invasiva (VNI)^(19)^, dos (16,7%) VNI mediante prongs binasales cortas^(25,28)^, uno (8,3%) la CPAP mediante prongs binasales y cánulas nasales de alto flujo calentadas y humidificadas, del inglés, *heated humidified high-flow nasal cannulae* (HHHFNC)^(16)^, uno (8,3%) una cánula nasal mediante CPAP y/o ventilación con presión positiva intermitente en las vías aéreas nasales^(27)^; uno (8,3%), prongs binasales mediante CPAP o ventilación con presión positiva intermitente nasal^(24)^; uno (8,3%), solo prongs para cánula nasal humidificada y calentada de alto flujo^(17)^; uno (8,3%) utilizó NIV mediante oxigenoterapia de alto flujo (OAF) y/o CPAP/*Bilevel Positive Airway Pressure* (BiPAP), presión positiva en dos niveles en las vías respiratorias, mediante cánula RAM®, varillas nasales, máscara nasal y gafas nasales^(18)^.

En cuanto al material intervencionista probado, en seis investigaciones (50%), los autores aplicaron el curativo de hidrocoloide^(16,17,20,23,24,26)^; en una (8,3%) se utilizó el de poliuretano con cloruro de polivinilo^(27)^; en dos (16,7%), el gel de silicona^(15,28)^ y, en uno (8,3%), los investigadores compararon el hidrocoloide con espuma de poliuretano con cloruro de polivinilo^(18)^. En otro estudio (8,3%), los investigadores analizaron protectores nasales de espuma fina e hidrocoloide^(19)^ y, en uno (8,3%)^(25)^, se comparó el hidrocoloide con gel de silicona de diferentes espesores. El tiempo de uso de las intervenciones y el seguimiento varió de menos de dos días^(28)^ a cuatro semanas^(17)^.

La mayoría de los estudios incluidos en esta revisión se realizaron con recién nacidos prematuros, la mayoría de ellos con una edad gestacional (EG) de 32 semanas o menos. El peso al nacer varió entre 825,8 y 2531,48 gramos.

De forma cronológica, el Cuadro 1 resume las características de los estudios que representaron el corpus de esta revisión.

**Cuadro 1:** Características de los estudios primarios incluidos en la revisión sistemática – Santa Maria, RS, Brasil, 2024.

Autor/año de publicación/muestra	Tipo de estudio	Grupo intervención (GI) (n)	Grupo control (GC) (n)	Dispositivo respiratorio	Síntesis de los principales resultados
Günlemez et al., 2010^(15)^ n = 179 neonatos prematuros	ECR	Lámina de gel de silicona (Epi-Derm Silicon Gel Sheeting, 1,8 mm de grosor, Biodermis) (n = 92)	Sin intervención (n = 87)	CPAP con prongs nasales	I: GI: 4,3% (n = 4) GC: 14,9% (n = 13) (OR: 3,43; IC 95%, 1,1–10,1; p < 0,05)
Xie, 2014^(23)^ n = 65 neonatos prematuros	ECR	Hidrocoloide (1,8 mm de espesor, 90029T, 3M Company) (n = 33)	Aceite de parafina (n = 32)	CPAP con prongs nasales	I: GI: 6,0% (n = 2) GC: 21,8% (n = 7) p = 0,01
Collins et al., 2014^(16)^ n = 132 neonatos prematuros	ECR	Grupo HHHFNC, todos utilizaron curativo Sticky Whiskers^®^. (hidrocoloide) (n = 67)	Grupo CPAP nasal: curativo Sticky Whiskers^®^ e Cannulaide^®^ (hidrocoloide) (n = 65)	CPAP por medio de prongs binasales y HHHFNC	Ausencia de datos sobre I La puntuación media de traumatismo nasal para los lactantes del grupo HHHFNC**** fue de 2,8 (DP 5,7), en comparación con 11,7 (DP 10,4) en el grupo CPAP** nasal (p < 0,001). No hubo diferencias en la puntuación media de traumatismo entre los recién nacidos en CPAP nasal que recibieron Sticky Whiskers^®^ (14,4; DE 12,5) o Cannulaide^®^ (9,5; DE 7,3), p = 0,06.
Morris et al., 2015^(17)^ n = 53 neonatos prematuros	Cohorte	Curativo hidrocoloide Com-feel Plus™ (Coloplast Corp.) (n = 26)	Sin curativo de barrera (n = 27)	HHHFNC	Ausencia de datos sobre I No hubo diferencias estadísticas en el estado de la piel de las fosas nasales entre los grupos (Wald χ^2^ = 1,79 df = 1, p = 0,18) ni a lo largo del tiempo (Wald χ^2^ = 2,7 df = 3, p = 0,45).
Badr et al., 2016^(27)^ n = 101 neonatos prematuros	Cohorte	*NeoSeal*®: Curativo de espuma de poliuretano con cloruro de polivinilo (PVC), espuma e adhesivo sensible a la presión (n = 51)	Sin curativo (n = 50)	CPAP mediante cánulas nasales y ventilación con presión positiva intermitente en las vías respiratorias nasales.	I: Grupo sin curativo: 24% (n = 12) Grupo *NeoSeal®*: 5,88% (n = 3) (OR = 4,08; 95% CI, 1,22–9,59; p = 0,01)
Imbulana et al., 2018^(24)^ n = 108 neonatos prematuros	ECR	Curativo de hidrocoloide NeoGuard (Readmed Inc, Jiangsu Province, China; Australian Register of Therapeutic Goods Identifier 160458 Class 1) (n = 53)	Sin curativo (n = 55)	Prongs binasales (CPAP y ventilación nasal con presión positiva intermitente)	I: GI: 34,0% (n = 18) GC: 56,4% (n = 31) (p = 0,02)
Ribeiro et al., 2019^(28)^ n = 11 neonatos	Caso-control	Curativo autoadhesivo de silicona gel de Mölnlycke Health Care’s Mepiform^®^ 0.3 mm de espesor. (n = 11)	VNI con prongs binasales curtas	I: 8 (72,7%)
Celda et al., 2020^(18)^ n = 13 neonatos	Cohorte	Grupo A: curativo de espuma de poliuretano *NeoSeal®*, con cloruro de polivinilo (PVC), espuma y adhesivo sensible a la presión (n = 7)	Grupo B: curativo hidrocoloide extrafino Comfeel Plus transparente (n = 6)	VNI (OAF), (CPAP/BiPAP) Interfaces: cánula RAM; varillas nasales; máscara nasal, gafas nasales	I: Grupo A: 40% (n = 2) Grupo B: 60% (n = 3) (χ^2^ = 0,627, *p* = 0,429)
Ribeiro et al., 2020^(25)^ n = 33 neonatos prematuros	ECR	Grupo A: hidrocoloide transparente (Comfeel Plus, Coloplast) (n = 11) Grupo B: lámina de gel de silicona espesa (Epi-Derm Silicone Gel Sheeting, Biodermis, 0,9 mm) (n = 11) Grupo C: silicona fina adherente (Mepiform, Molnlycke Health Care) (n = 11)	No se aplica	VNI con prongs bi-nasales cortas	I: Grupo A: 36,36% (n = 4) Grupo B: 81,81% (n = 9) Grupo C: 72,72% (n = 8) (p = 0,06)
Yang et al., 2021^(20)^ n = 106 neonatos prematuros	ECR	Hidrocoloide (n = 53)	Plug nasal en formato de U, o tipo de material no fue informado (n = 53)	CPAP nasal	I: GI: 15% (n = 8) GC: 52,8% (n = 28) (p < 0,05)
Rezaei et al., 2021^(26)^ n = 80 neonatos prematuros	ECR	Curativo de hidrocoloide (Neo-Guard; ConLett) (n = 40)	Cuidados rutinarios (masaje del tabique nasal y las fosas nasales con pomada oftálmica de cloruro sódico al 5%) (n = 40)	CPAP con prongs nasales	I: GI: 37,5% (n = 15) GC: 92,5% (n = 37) (p < 0,001)
Nguyen et al., 2021^(19)^ n = 113 neonatos prematuros	Cohorte	Curativo hidrocoloide (n = 71)	Protectores nasales de espuma fina de 1 mm. Tipo de material no informado (n = 42)	Cánula nasal para VNI	Prevalencia de lesiones por presión nasal Grupo hidrocoloide: 2,8% (n = 2) Grupo espuma fina: 23,8% (n = 10) (OR = 0,09, IC del 95%, 0,02-0,45) (p = 0,001)

Notas: ECR: ensayo clínico randomizado; CPAP: continuous positive airway pressure (presión positiva continua en las vías respiratorias nasales); I: incidencia de lesiones por presión nasal; HHHFNC: heated humidified high-flow nasal cannulae (cánula nasal de alto flujo calentada y humidificada); VNI: ventilación no invasiva; OAF: oxigenoterapia de alto flujo; BiPAP bilevel positive airway pressure: presión positiva de dos niveles en las vías respiratorias (presión positiva en dos niveles en las vías respiratorias).

### Calidad Metodológica de los Estudios

Se utilizaron las herramientas propuestas por la Colaboración JBI para la evaluación metodológica de los estudios. Cabe destacar que ninguna de ellas cuenta con un sistema de puntuación para la evaluación general de la investigación, pero un mayor número de respuestas “sí” es indicativo de una mejor calidad metodológica.

Así, mediante la herramienta *JBI Critical Appraisal Checklist for Randomized Controlled Trial* ^(9)^ (n = 7) (Cuadro 2), de las 13 preguntas que componen la lista de verificación, en tres investigaciones^(16,24,26)^, once preguntas recibieron la respuesta “sí”; en dos^(15,20)^, nueve preguntas y, en dos estudios^(23,25)^, ocho preguntas. La pregunta sobre el enmascaramiento de quienes administraron el tratamiento (en este caso, aplicaron los curativos) recibió la respuesta “no” en todos los estudios. En todas las investigaciones, las respuestas fueron “sí” a las preguntas sobre la similitud entre los grupos de intervención y control; el enmascaramiento de los participantes en relación con la intervención; la administración de la intervención; el seguimiento; la medición de los resultados y el uso de análisis estadísticos adecuados.

**Cuadro 2:** JBI Critical Appraisal Checklist for Randomized Controlled Trial – Santa Maria, RS, Brasil, 2024.

Referencia	Pregunta												
	1	2	3	4	5	6	7	8	9	10	11	12	13
Günlemez et al., 2010^(15)^	N	N	S	S	N	S	S	S	S	S	S	S	N
Xie, 2014^(23)^	N	N	S	S	N	N	S	S	S	S	S	S	N
Collins et al., 2014^(16)^	S	S	S	S	N	N	S	S	S	S	S	S	S
Imbulana et al., 2018^(24)^	S	S	S	S	N	S	S	S	NC	S	S	S	S
Ribeiro et al., 2020^(25)^	N	N	S	S	N	N	S	S	S	S	S	S	N
Yang et al., 2021^(20)^	S	N	S	S	N	N	S	S	S	S	S	S	N
Rezaei et al.*,* 2021^(26)^	S	S	S	S	N	N	S	S	S	S	S	S	S
**Porcentaje (%)**	57	43	100	100	0	28	100	100	85	100	100	100	43
P1: ¿Se utilizó una randomización verdadera para asignar a los participantes a los grupos de tratamiento? P2: ¿Se ocultó la asignación de los grupos? P3: ¿Los grupos de tratamiento eran similares en la línea de base? P4: ¿Los participantes estaban cegados respecto a la asignación del tratamiento? P5: ¿Los que administraron el tratamiento estaban cegados respecto a la asignación del tratamiento? P6: ¿Los evaluadores de resultados estaban cegados respecto a la asignación del tratamiento? P7: ¿Los grupos de tratamiento recibieron un tratamiento idéntico, salvo por la intervención de interés? P8: ¿El seguimiento fue completo y, en caso contrario, se describieron y analizaron adecuadamente las diferencias entre los grupos en relación con el seguimiento? P9: ¿Se analizó a los participantes en los grupos a los que fueron asignados aleatoriamente? P10: ¿Se midieron los resultados de la misma manera para los grupos de tratamiento? P11: ¿Se midieron los resultados de manera confiable? P12: ¿Se utilizó un análisis estadístico adecuado? P13: ¿El diseño del estudio fue adecuado y se tuvieron en cuenta en la realización y el análisis cualquier desviación del diseño estándar de los ECR?													

Abreviatura: S = si; N = no; NC = no claro. Fuente: autores; JBI^(9)^.

Al aplicar la herramienta *JBI Critical Appraisal Checklist for Cohort Studies* ^(9)^ (n = 4), de las 10 preguntas que componen la lista de verificación, con opciones de respuesta “sí”, “no”, “no está claro” y “no se aplica”, la investigación^(17)^ recibió una respuesta “no” a cuatro preguntas sobre los factores de confusión y las estrategias para abordarlos, sobre el seguimiento completo y las estrategias para abordar el seguimiento incompleto; el estudio^(27)^ recibió una respuesta “no” solo a la pregunta sobre las estrategias para abordar el seguimiento incompleto; la investigación^(18)^ recibió una respuesta “no” a las preguntas sobre el tiempo de seguimiento y las estrategias para abordar el seguimiento incompleto; el estudio^(19)^ recibió una respuesta “no” a las preguntas sobre el tiempo de seguimiento, la descripción de los motivos de las pérdidas de seguimiento y las estrategias para abordar el seguimiento incompleto.

Las preguntas dirigidas al reclutamiento de los grupos en relación con la exposición, para la medición válida y confiable de las exposiciones, para el análisis de los participantes, si estaban libres de los desenlaces de interés al inicio del estudio y para el método estadístico empleado recibieron una respuesta “sí” en los cuatro estudios. La pregunta sobre las estrategias para abordar el seguimiento incompleto no se contempló en ninguno de los estudios incluidos^(17–19,27)^.

La herramienta *Critical Appraisal Checklist for Case-Control Studies* ^(9)^ (n = 1) contiene 10 preguntas. El único estudio de casos y controles incluido^(28)^ recibió una respuesta “no” en tres ítems relacionados con la identificación y el manejo de factores de confusión y el análisis estadístico utilizado. Las otras siete preguntas, que abordan la comparación entre grupos, la combinación adecuada de casos y controles, los criterios de identificación, la medición de la exposición, la evaluación estandarizada y confiable de los resultados y el período de exposición, recibieron una respuesta “sí”.

### Metaanálisis en Red

El metaanálisis en red para el resultado de la ocurrencia de LP nasal se compuso de 571 recién nacidos prematuros, y los datos se extrajeron de seis ensayos clínicos aleatorios^(15,20,23-26)^. Un estudio^(16)^ no se incluyó porque, a pesar de cumplir con los criterios de elegibilidad de la revisión, no se presentaron los datos sobre la ocurrencia del evento en frecuencia absoluta o relativa, lo que imposibilitó su inclusión en los modelos cuantitativos de este metaanálisis de datos dicotómicos (se solicitaron los datos, pero los autores no respondieron).

La estructura de las intervenciones que permitieron el metaanálisis en red se presenta gráficamente en la Figura 2, una red de ensayos clínicos que comparan las intervenciones para la prevención de la LP nasal en recién nacidos que utilizaron dispositivos médicos respiratorios. El ancho de los bordes es proporcional al número de pacientes aleatorizados en cada comparación. Los colores de los bordes y los nodos se refieren al riesgo de sesgo, siendo: bajo, en verde; moderado, en amarillo. Además, los círculos representan las intervenciones y las líneas rectas son las comparaciones directas. Cuanto mayor es el grosor, mayor es el número de estudios que realizaron una determinada comparación.

**Figura 2:** Geometría de la red de posibles intervenciones para realizar el metaanálisis en red obtenido en el *software Webapp Confidence in Network Meta-Analysis*.

En la Tabla 1 se presentan las comparaciones directas e indirectas de los resultados sobre la eficacia de los curativos profilácticos para la aparición de LP nasal en neonatos prematuros que utilizan dispositivos médicos respiratorios. Estos resultados demostraron que hubo una diferencia significativa entre las intervenciones.

**Tabla 1:** Tabla de ligas (*league table*) de comparaciones directas e indirectas de los resultados sobre la eficacia de los curativos profilácticos para la aparición de lesiones por presión nasal en neonatos prematuros que utilizan dispositivos médicos respiratorios, en diferentes grupos – Santa Maria, RS, Brasil, 2024.

Cuidados de Rutina	1,09 (0,43, 2,76)	**5,10 (1,48, 17, 61)**	**2,46 (1,63, 3,71)**	0,68 (0,14, 3,12)	0,70 (0,31, 1,56)	1,48 (0,81, 2,71)	1,23 (0,47, 3,20)
	Gel de Silicona 0,9 mm	**4,65 (1,11, 19,53)**	2,25 (0,98, 5,15)	0,62 (0,11, 3,44)	0,64 (0,21, 1,88)	1,35 (0,53, 3,47)	1,12 (0,71, 1,77)
		Gel de Silicona 1,8 mm	0,48 (0,15, 1,55)	**0,13 (0,02, 0,89)**	**0,13 (0,03, 0,53)**	**0,29 (0,09, 0,85)**	0,24 (0,05, 1,03)
			Hidrocoloide	0,27 (0,62, 1,23)	**0,28 (0,14, 0,56)**	**0,60 (0,38, 0,93)**	0,50 (0,21, 1,18)
				Aceite de Parafina	1,03 (0,19, 5,34)	2,17 (0,45, 10,33)	1,80 (0,32,10,12)
					Plug Nasal U	2,10 (0,93, 4,77)	1,75 (0,58, 5,26)
						Sin Intervención	0,83 (0,31, 2,18)
							Silicona Fina Adherente

Notas: Los cuidados rutinarios, el aceite de parafina, los plugs nasales, la silicona fina adherente y la ausencia de intervención son comparadores comunes. Las comparaciones entre las intervenciones deben interpretarse de izquierda a derecha y de arriba abajo. Para el metaanálisis de pares (esquina superior derecha) se utilizó el RR (riesgo relativo), con sus respectivos intervalos de confianza (IC). Fuente: autores; CINeMA, 2024.

Se observó que los cuidados rutinarios aumentan el riesgo de aparición de LP nasal, en comparación con el gel de silicona de 1,8 mm (RR 5,10; IC 95% = 1,48, 17,61) y el hidrocoloide (RR 2,46; IC 95% = 1,63, 3,71), así como el gel de silicona de 0,9 mm en comparación con el de 1,8 mm (RR 4,65; IC 95% = 1,11, 19,53). También se observó que el uso de silicona de 1,8 mm reduce el riesgo de aparición de LP nasal, en comparación con el aceite de parafina (RR 0,13; IC del 95% = 0,02, 0,89), el plug nasal (RR 0,13; IC del 95% = 0,03, 0,53) y la no intervención (RR 0,29; IC del 95% = 0,09, 0,85), así como que el uso de hidrocoloide reduce el riesgo de lesión en comparación con el plug (RR 0,28; IC del 95% = 0,14, 0,56) y la no intervención (RR 0,60; IC del 95% = 0,38, 0,93).

### Certeza de la Evidencia

La evaluación realizada por CINeMA señaló una certeza de evidencia “baja” para el resultado de aparición de LP nasal en neonatos que utilizan dispositivos respiratorios, teniendo en cuenta las intervenciones y los comparadores evaluados.

## DISCUSIÓN

Los resultados de la revisión demostraron los beneficios de utilizar curativos profilácticos para prevenir la aparición de LP nasal en neonatos prematuros que necesitan asistencia respiratoria, indicando diferentes formas de intervenir. Aunque se incluyeron estudios con diferencias metodológicas, fue posible construir una síntesis de evidencia sólida que puede contribuir a la mejora de la práctica clínica.

Aunque no estaba previsto, todos los estudios incluidos en el metaanálisis en red se realizaron con una población de recién nacidos prematuros. Esto se debe a que la insuficiencia respiratoria relacionada con la inmadurez pulmonar es una de las morbilidades inmediatas más comunes entre los prematuros, muchos de los cuales desarrollan complicaciones a largo plazo^(29)^.

En las investigaciones evaluadas, predominaron los recién nacidos que necesitaron CPAP y VNI. El uso de VNI y CPAP nasal para el soporte respiratorio de los recién nacidos prematuros se ha asociado con una reducción modesta en la incidencia de daño pulmonar, previniendo la progresión al síndrome de dificultad respiratoria, especialmente si se inicia de forma profiláctica, antes de que se manifieste la atelectasia^(30)^.

El metaanálisis en red indicó que los cuidados de rutina y el uso de gel de silicona de 0,9 mm no contribuyen a disminuir la incidencia de LP nasal en neonatos prematuros. Por otro lado, este resultado se previene cuando se utiliza silicona de 1,8 mm o hidrocoloide.

En un estudio, cuyos autores utilizaron un enfoque experimental-computacional integrado de un individuo adulto, se comparó el rendimiento de la protección biomecánica de tres curativos a base de espuma, uno de ellos con silicona, con el de hidrocoloide para proteger la piel facial bajo una mascarilla de CPAP. En la zona nasal, la diferencia en el cálculo del índice de eficacia protectora fue del 86% para el apósito de espuma con silicona, frente al 60% para el hidrocoloide^(4)^.

Desde esta perspectiva, la prevención de la LP nasal en recién nacidos que necesitan asistencia respiratoria es un reto multifactorial. Aunque el uso de curativos profilácticos desempeña un papel importante en la prevención de este tipo de lesiones, y aunque se ha demostrado su eficacia para reducir este resultado, hay que tener en cuenta otros factores. La duración de la terapia ventilatoria, el tipo de dispositivo utilizado y sus interfaces están directamente relacionados con la etiología de este daño tisular^(18,26)^. Además, las características del recién nacido, como el peso al nacer y la edad gestacional, son determinantes significativos en el aumento del riesgo de esta complicación^(19)^.

La aparición de LP aumenta los costos hospitalarios debido al manejo de complicaciones secundarias. En una revisión sistemática, los autores evaluaron la incidencia, la prevalencia, el tiempo de hospitalización atribuible y el costo de la LP adquirida en el hospital en pediatría, incluidos los recién nacidos, y los resultados indicaron una prevalencia del 27,0% (IC del 95%: 22,1% – 33,1%) y una incidencia del 9,8% (IC del 95%: 2,9% – 19,8%). Para la población pediátrica en general, la duración de la hospitalización varió de 0,9 a 14,1 días, y el costo atribuible varió de 894,69 a 98 730,24 dólares estadounidenses (valor del dólar en 2020) por paciente con LP nosocomiales^(31)^. Por lo tanto, las estrategias preventivas, como el uso de estas cubiertas protectoras, pueden generar ahorros a largo plazo al reducir la incidencia de lesiones y los costos de hospitalización, además de minimizar los daños evitables, alineándose con los principios de seguridad del paciente y buenas prácticas en neonatología.

Al aplicar los instrumentos de evaluación de la calidad metodológica para ensayos clínicos aleatorios, se pudo analizar que en algunos estudios no se presentaron datos sobre la aleatorización y la asignación^(15,23,25>)^, así como aquellos que administraban los curativos^(15,20,23–26)^ y que evaluaban el resultado no estaban cegados^(16,20,23,25,26)^. Sin embargo, en estos estudios, el enmascaramiento de los profesionales y evaluadores es inviable debido a la propia naturaleza de la intervención y requiere una aplicación que el profesional debe conocer, como es el caso de los curativos. Esto implica un sesgo relacionado con la selección y la asignación, la administración de la intervención/exposición y la evaluación, detección y medición del resultado, lo que puede comprometer la validez interna de estas investigaciones^(32)^.

En dos estudios observacionales incluidos en la revisión, los resultados demostraron que la intervención (curativo profiláctico) provocó una reducción de la PL nasal en los recién nacidos que utilizaban equipos de terapia ventilatoria, lo que indica el beneficio de su aplicación^(19,27)^. Sin embargo, en los otros tres estudios, los resultados no demostraron los beneficios de la intervención, ya que no evidenciaron mejoras en la prevención de esta complicación^(17,18,28)^. Estas variaciones e inconsistencias entre los resultados de los estudios observacionales pueden estar relacionadas con posibles sesgos identificados durante la aplicación de los instrumentos de evaluación de la calidad metodológica, ya que algunos no presentaron la identificación de factores de confusión ni la declaración de estrategias para lidiar con ellos. Además, algunos no describieron el seguimiento completo ni los motivos de las pérdidas y no presentaron estrategias para lidiar con el seguimiento incompleto^(17–19,27)^.

La certeza de la evidencia clasificada como “baja” indica una confianza limitada en el efecto, que puede verse influida por nuevas investigaciones, aspecto que puede atribuirse a la presencia de sesgos de selección en algunos de los estudios incluidos, relacionados con la falta de aleatorización adecuada y la falta de ocultación de la asignación; también por la presencia de diferencias en las intervenciones entre los estudios incluidos, en cuanto al tipo de material y la falta de estandarización en los comparadores, además de la limitación en las muestras y el número de eventos tanto en el grupo de intervención como en los comparadores.

Como implicación para la investigación, a fin de reforzar las recomendaciones futuras con el propósito de implementar la práctica basada en la evidencia en la práctica clínica, se destaca la necesidad de realizar estudios con una metodología sólida, con muestras representativas, un control riguroso del sesgo y la estandarización de las intervenciones investigadas, incluyendo criterios consistentes para la evaluación de los resultados. Además, en futuros estudios deben tenerse cuidadosamente en cuenta factores como la duración y el tipo de dispositivo respiratorio y sus interfaces, así como las características del recién nacido, como el peso al nacer y la edad gestacional.

En este contexto, la selección de barreras protectoras cutáneas eficaces tiene un impacto en los resultados clínicos y en los costos asociados a la atención sanitaria. Por lo tanto, es importante comprender mecanismos como la capacidad del apósito para reducir los gradientes de rigidez entre los materiales de los equipos y la piel. El apóste debe favorecer el aumento del área de contacto entre los contornos del dispositivo y la superficie cutánea, permitiendo la redistribución de las cargas. Estas propiedades son fundamentales para reducir las concentraciones de tensión en la piel y los tejidos subyacentes, contribuyendo a la prevención de este daño tisular^(33)^.

Como implicación para la práctica, los resultados del metaanálisis en red respaldan la indicación del uso de curativos profilácticos de hidrocoloide y gel de silicona de 1,8 mm como intervenciones eficaces para prevenir la LP nasal en neonatos prematuros, entre la superficie de las fosas nasales y la interfaz del equipo respiratorio. Para este fin, los curativos pueden recortarse con la forma de la base nasal, con dos orificios adaptados a la anatomía de la nariz y las fosas nasales, lo que permite un posicionamiento adecuado del dispositivo y favorece la protección de la piel^(20,23,25>)^.

Así, las evidencias generadas en la realización de esta revisión pueden respaldar la toma de decisiones de los profesionales de la salud, sugiriendo que la inclusión de curativos profilácticos como parte de la rutina de cuidado de la piel neonatal puede mejorar la seguridad del paciente y la calidad de la atención prestada a esta población vulnerable.

### Limitaciones del Estudio

Si bien los metaanálisis constituyen la mejor evidencia, los datos de la presente revisión deben interpretarse teniendo en cuenta las particularidades de los estudios primarios incluidos. Además, los datos no encontrados en algunos de los estudios incluidos, la heterogeneidad de las intervenciones probadas y la forma de evaluar la aparición de LP nasal limitaron otras comparaciones.

## CONCLUSIÓN

La presente revisión identificó evidencia favorable al uso de curativos profilácticos en la prevención de la LP nasal en recién nacidos prematuros que utilizan dispositivos de soporte respiratorio no invasivo. Los apósesos de hidrocoloide y de gel de silicona con un espesor de 1,8 mm se destacaron como intervenciones eficaces para mitigar esta afección. Sin embargo, la calidad metodológica de los estudios incluidos en el metaanálisis en red dio como resultado un nivel de certeza de la evidencia clasificado como bajo, lo que configura limitaciones para la generalización de los hallazgos y exige cautela en su incorporación a la práctica clínica.

Aun así, los resultados de esta revisión sistemática proporcionan información útil para la práctica asistencial neonatal, sugiriendo el uso criterioso de curativos profilácticos como estrategia preventiva adyuvante en las UCIN. Sin embargo, cabe destacar la necesidad de nuevos ECR, con diseños metodológicos consistentes, a fin de consolidar la evidencia científica sobre la eficacia y seguridad de estas intervenciones. Así, este estudio contribuye al conjunto de conocimientos existentes, ya que delinea las lagunas que orientan futuras investigaciones en el área.

## Data Availability

El conjunto de datos que respalda los resultados de este estudio no está disponible públicamente.
